# Near-Infrared Fluorescent Probe for the In Situ Visualization of Oxidative Stress in the Brains of Neuroinflammatory and Schizophrenic Mice

**DOI:** 10.1021/acs.analchem.3c01447

**Published:** 2023-08-01

**Authors:** Yujie Geng, Hanchen Zhang, Guoyang Zhang, Jiaying Zhou, Mingguang Zhu, Lijun Ma, Xuefei Wang, Tony D. James, Zhuo Wang

**Affiliations:** † State Key Laboratory of Chemical Resource Engineering, College of Chemistry, Beijing Advanced Innovation Center for Soft Matter Science and Engineering, Beijing University of Chemical Technology, Beijing 100029, P. R. China; ‡ School of Chemical Science, University of Chinese Academy of Sciences, Beijing 100049, P. R. China; § Department of Chemistry, University of Bath, Bath BA2 7AY, U.K.; ∥ School of Chemistry and Chemical Engineering, Henan Normal University, Xinxiang 453007, P. R. China; ⊥ Institute of Chemistry, Chinese Academy of Sciences, Zhongguancun North First Street 2, 100190 Beijing, P. R. China

## Abstract

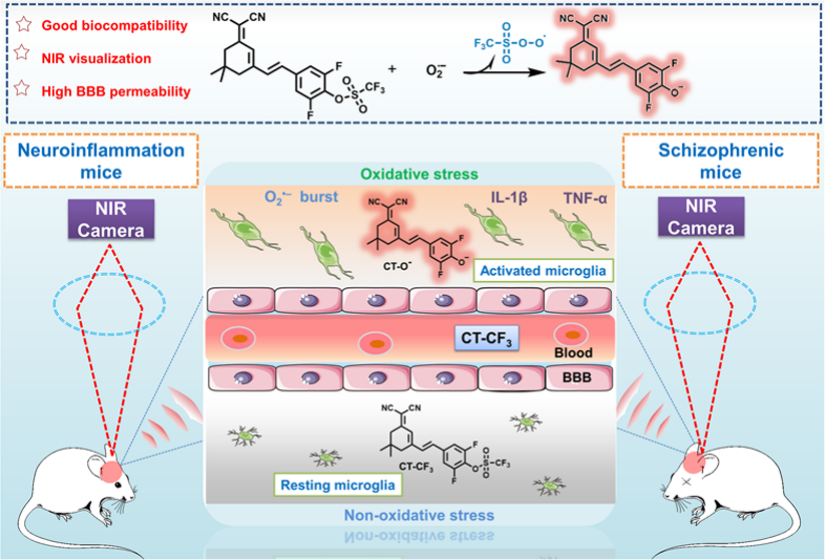

Schizophrenia is a common mental disorder with unclear mechanisms. Oxidative stress and neuroinflammation play important roles in the pathological process of schizophrenia. Superoxide anion (O_2_^•–^) is an important oxidative stress biomarker *in vivo*. However, due to the existence of the blood–brain barrier (BBB), few near-infrared (NIR) fluorescent probes have been used for the sensing and detection of O_2_^•–^ in the brain. With this research, we developed the first near-infrared fluorescent probe (named CT–CF_3_) for noninvasive detection of endogenous O_2_^•–^ in the brain of mice. Enabling fluorescence monitoring of the dynamic changes in O_2_^•–^ flux due to the prolonged activation of microglia in neuroinflamed and schizophrenic (SZ) mice brains, thereby providing direct evidence for the relationship between oxidative stress, neuroinflammation, and schizophrenia. Furthermore, we confirmed the O_2_^•–^ burst in the brains of first-episode schizophrenic mice and assessed the effect of two atypical antipsychotic drugs (risperidone and olanzapine) on redox homeostasis.

Schizophrenia is a serious chronic mental illness that occurs mostly in late adolescence and early adulthood.^1^ The symptoms of this disease are mainly divided into three categories: positive symptoms (delusions, hallucinations, etc.), negative symptoms (affective flattening and deficits in social function), and cognitive deficits.^2^ Currently, nearly 1% of the world’s population suffers from schizophrenia, placing a heavy burden on the global economy and health care systems.^3^ Due to the complexity of the disease, the etiology, pathogenesis, and biological processes of schizophrenia are not fully understood. However, it is known that neuroinflammation and oxidative stress play crucial roles in the pathophysiology of schizophrenia.^4^

The excessive production of oxidants and the severe imbalance of antioxidant consumption in organisms are defined as oxidative stress.^5^ Previous research has indicated increased oxidative damage of lipids, proteins, and DNA and decreased glutathione levels in the brain tissue and blood of schizophrenia patients.^6−9^ The behavioral and molecular anomalies induced by oxidative stress in animal models are similar to those in patients with schizophrenia.^10^ Neuroinflammation is central to the common pathology of many psychiatric disorders.^11^ Excessive and long-term neuroinflammation is closely associated with the pathology of schizophrenia and other brain diseases. SZ patients are in a chronic inflammatory state, resulting in a decrease in anti-inflammatory cytokines and an increase in inflammatory cytokines.^12,13^ Oxidative stress and inflammation are mutually reinforcing. Inflammation induces abnormally elevated levels of oxidants, and oxidative stress also induces inflammation through activation of nuclear factor kappa B (NF-κB).^4^ Therefore, the precise monitoring of oxidative stress will facilitate a better understanding of the etiology, pathogenesis, and biological processes of schizophrenia. Superoxide anion (O_2_^•–^) is the one-electron reduction product of O_2_ and plays an important role in regulating cellular signaling networks.^14^ Due to the short half-life (10^–6^ s) and high reactivity, real-time monitoring of O_2_^•–^*in vivo* is essential. In recent times, fluorescent probes have become widely accepted as tools to evaluate abnormal fluctuations of O_2_^•–^ levels in living cells and *in vivo*.^15^ However, due to the existence of the blood–brain barrier (BBB), few fluorescent probes have been used for the detection of O_2_^•–^ in the living brain. The BBB blocks about 98% of small molecules and almost all macromolecules and effectively protects neurons from harmful substances in the blood.^16,17^ Several structural design strategies are known to assist small molecules cross the BBB more easily. General principles include the following: the lipid–water partition coefficient (Log *P*) should be between 2 and 5, the molecular weight should be less than 500 Da, the molecules should exhibit weak hydrogen bonding capability, with less than 3 hydrogen bond donors, the molecular flexibility and rotatable bonds should be limited.^18−20^

Dicyanoisophoron (CN–OH) is a popular near-infrared fluorophore and has been widely used in fluorescent probe design for disease marker identification.^21−23^ In this work, we synthesized three near-infrared fluorophores based on dicyanoisophorone. Among them, CT–OH was used as the fluorophore due to its excellent p*K*_a_ (5.68). Furthermore, we excluded some of the commonly used O_2_^•–^ recognition groups that have large molecular weights or include many hydrogen bond donors, such as catechol, 2,4-dinitrobenzenesulfonyl, and the diphenylphosphinate. Therefore, the trifluoromethanesulfonate group was used as the recognition group for O_2_^•–^. The probe CT–CF_3_ exhibited good efficiency in crossing the blood–brain barrier (transport: 2.52% ID/g at 5 min). As such, CT–CF_3_ could be used for the first time to demonstrate the overproduction of endogenous O_2_^•–^ in the brains of neuroinflammatory mice and SZ mice due to prolonged activation of microglia and assess the changes of O_2_^•–^ levels in the brains of first-episode schizophrenic mice before and after drug treatment.

## Experimental Section

### Synthesis of Probe

#### Synthesis of CN–OH and CF–-OH

The procedure for the synthesis of CN–OH and CF–-OH is described in the Supporting Information.

#### Synthesis of CT–OH

Compound 1 (37.2 mg, 0.2 mmol) and 3,5-Difluoro-4-hydroxybenzaldehyde (31.6 mg, 0.2 mmol) were added to a solution of ethanol (10 mL) containing a catalytic amount of piperidine and refluxed under N_2_ atmosphere for 6 h. Then, the mixture was evaporated under reduced pressure. The residue was purified by column chromatography (petroleum ether: CH_2_Cl_2_ = 1:1) to obtain an orange solid (23.4mg, 35.8%), ^1^H NMR (400 MHz, Methanol-*d*_4_) δ 7.25 (dd, *J* = 7.9, 1.8 Hz, 2H), 7.15–7.03 (m, 2H), 6.87 (s, 1H), 2.65 (s, 2H), 2.55 (s, 2H), 1.10 (s, 6H). ^13^C NMR (101 MHz, Methanol-*d*_4_) δ 169.71, 154.69, 153.84, 153.77, 151.43, 151.36, 135.28, 128.61, 127.31, 127.22, 127.14, 122.81, 113.16, 112.40, 110.63, 110.56, 110.48, 77.45, 42.52, 26.70. HR-MS (ESI, negative) calcd for C_19_H_15_F_2_N_2_O^–^, [M – H]^−^ 325.11579; found, 325.11584.

#### Synthesis of CT–CF_3_

As shown in Figure S2, the probe CT–CF_3_ was synthesized using CT–OH (0.0326 g, 0.1 mmol) and trifluoromethanesulfonic anhydride (0.11 mmol) in a mixture of pyridine (5 mL) and CH_2_Cl_2_ (5 mL) under the N_2_ atmosphere. The mixture was stirred at room temperature for 3 h. Then, the solvent was removed by evaporation under reduced pressure. The residue was purified by silica gel chromatography using CH_2_Cl_2_/petroleum ether (v/v, 1:3) as an eluent to get a pale-yellow product (0.032 g, 70.2%). ^1^H NMR (400 MHz, Chloroform-*d*) δ 7.22 (d, *J* = 8.3 Hz, 2H), 7.02–6.87 (m, 3H), 2.65 (s, 2H), 2.48 (s, 2H), 1.12 (s, 6H). ^13^C NMR (101 MHz, Chloroform-*d*) δ 168.17, 156.66, 153.75, 151.99, 137.49, 133.15, 132.26, 131.67, 125.05, 119.74, 116.29, 112.93, 112.17, 111.27, 110.75, 81.10, 42.90, 39.16, 32.05, 28.56. HR-MS (ESI, negative) calcd for C_20_H_14_F_5_N_2_O_3_S^–^, [M – H]^−^ 457.06507; found, 457.06494.

### Neuroinflammation and Schizophrenia Mouse Model

#### Preparation of an LPS-induced neuroinflammation mouse model

All mice were divided into three groups: (i) Control mice received daily intraperitoneal injections of PBS (5 mL/kg) depending on their body weight; (ii) In accordance with a previous mouse model of neuroinflammation,^24^ the experimental group of mice was modeled by intraperitoneal injection of LPS (0.25 mg/kg, 5 mL/kg for 7 days); (iii) After the same dose of LPS (0.25 mg/kg, 5 mL/kg for 7 days), N-acetylcysteine (NAC, a widely used antioxidant) was used to scavenge reactive oxygen species from the brain (20 mg/kg, 5 mL/kg for days 3 to 8) in the treatment group. The injection time was set between 14:00 and 15:00. The body weights of the three groups of mice were monitored daily, and the mice in the experimental and treatment groups experienced a sharp drop and a slow recovery (Figure S10B).

#### Preparation of a schizophrenia mouse model and fluorescence imaging

All mice were divided into three groups: (i) Control mice received daily intraperitoneal injections of PBS (5 mL/kg) depending on their body weight; (ii) Mice in the experimental group were modeled by intraperitoneal injection of MK-801 (0.6 mg/kg, 5 mL/kg for 14 days); (iii) Mice in the treatment group were modeled by intraperitoneal injection of MK-801 (0.6 mg/kg, 5 mL/kg for 14 days), together with a commonly used atypical antipsychotic, Olanzapine-treated (1 mg/kg, 5 mL/kg, days 12–14). The injection time was between 14:00 and 15:00.

## Results and Discussion

### Design and Synthesis of CT–CF_3_

Most of the current fluorescent probes for the detection of reactive oxygen species (ROS) in the brain use short-wavelength emitting two-photon probes.^25−27^ However, two-photon imaging requires the opening of a cranial window in the head of a living mouse, and such an invasive operation is really not friendly for bioimaging analysis. Near-infrared fluorescent probes with low background fluorescence and high tissue penetration are ideal tools for ROS detection in the brain.

**Figure 1:**
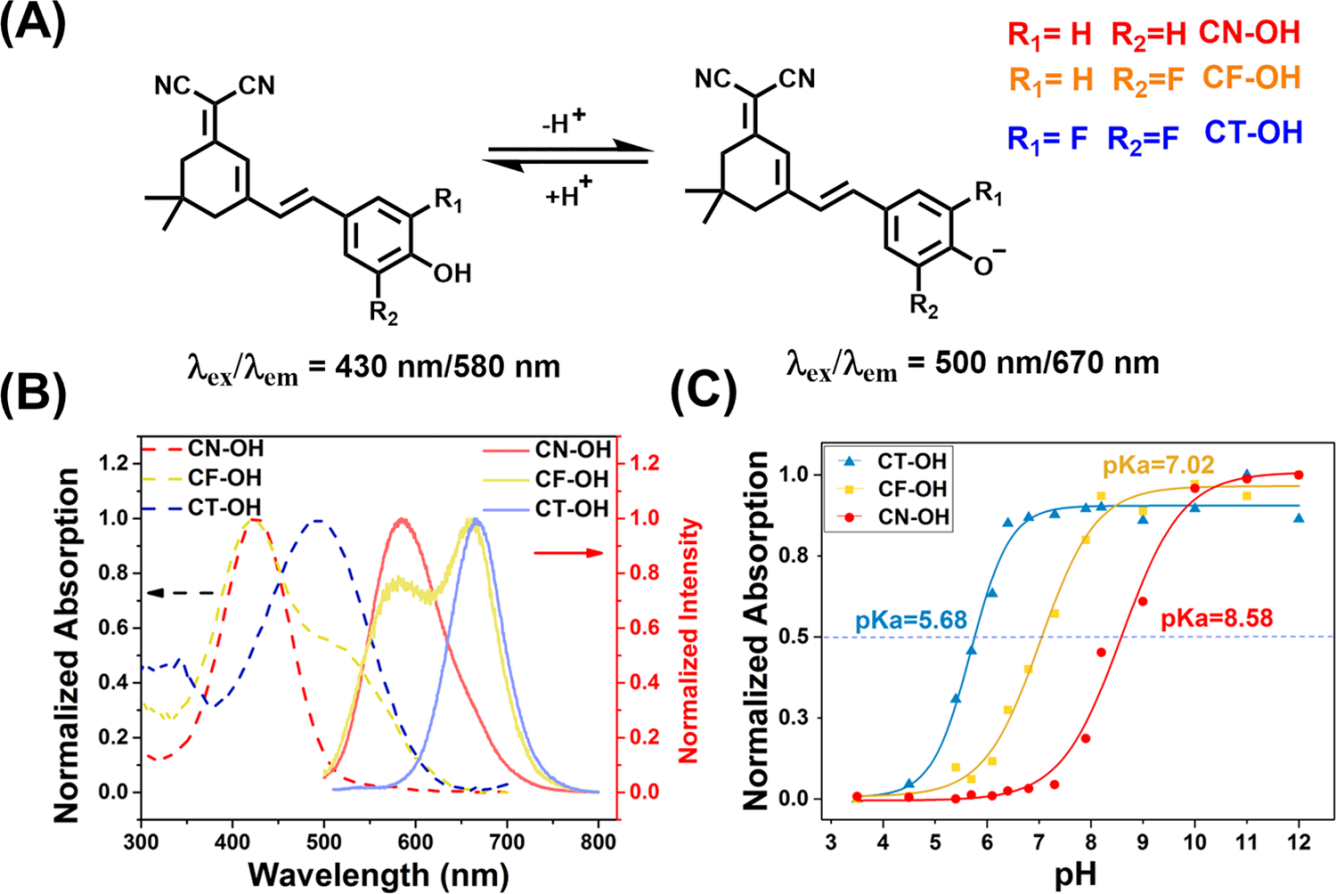
(A) Structural and spectral variations of three dicyanoisophorone systems in anionic and phenolic hydroxyl forms; (B) Absorption and emission wavelengths of CN–OH, CF-OH, and CT–OH in buffer solution (pH = 6.8, 30%DMSO); (C) Plots of absorption intensity at 500 nm as a function of pH.

We constructed the near-infrared fluorophore CN–OH based on dicyanoisophorone as the fluorescent framework (Figure 1A). Given that the physiological pH in the brain of SZ patients is slightly lower than that of healthy individuals,^28−30^ we adjusted the p*K*_a_ of the fluorophore by introducing one fluorine (CF-OH) and two fluorine atoms (CT–OH) at the ortho positions of the phenolic hydroxyl group, respectively (Figure 1A,B). In a weakly acidic buffer solution (pH = 6.8), CN–OH existed as a phenol with maximum absorption and emission wavelengths of 430 and 580 nm, respectively; CF-OH coexisted as both phenol and phenolic anions, so there were two absorption peaks at 430 and 500 nm and two emission peaks at 580 and 670 nm. In contrast, CT–OH is present exclusively as the phenolic anion, with maximum absorption and emission wavelengths of 500 nm and 670 nm, respectively. As shown in Figure 1C, the p*K*_a_ of the three fluorophores were 8.58, 7.02, and 5.68, respectively. To avoid interference with imaging accuracy from reduced physiological pH in the SZ brain, CT–OH was chosen as the fluorophore. While the trifluoromethanesulfonyl group served as a specific recognition group for O_2_^•–^, owing to its low molecular weight and lipid solubility.^31^

### Spectral Characterization

**Figure 2:**
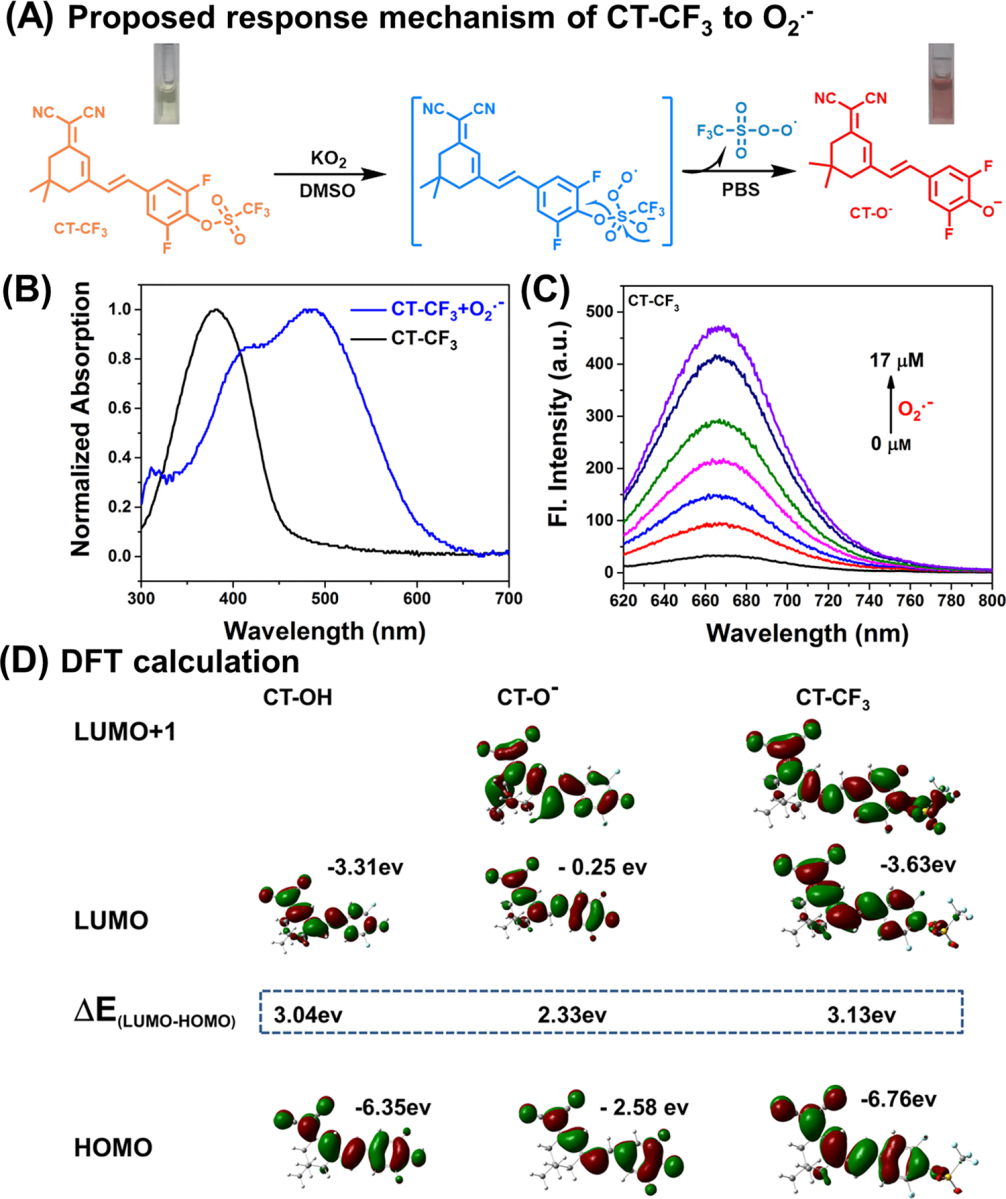
(A) Schematic diagram of CT–CF_3_ response to O_2_^•–^; (B) absorption spectra of probe CT–CF_3_ (5 μM) with and without O_2_^•–^ (100 μM) in PBS (pH = 7.4, 30% DMSO as co-solvent); (C) fluorescence spectra of 5 μM CT–CF_3_ and 0–17 μM O_2_^•–^ in PBS (pH = 7.4, 30% DMSO as co-solvent); (D) comparison of the frontier orbitals of the CT–OH, CT-O^–^, and CT–CF_3_ configuration and corresponding energy levels.

Through the esterification of trifluoromethanesulfonic anhydride and phenolic hydroxyl groups, we successfully obtained CT–CF_3_ (Figure S2). We then evaluated the optical properties and responsiveness of CT–CF_3_. KO_2_ decomposes rapidly in aqueous environments to form HO^–^ and HO_2_^–^ while KO_2_ can be stabilized in DMSO and produce O_2_^•–^.^32^ According to the previously reported protocols,^33^ KO_2_ (a dilute solution in DMSO) was added to CT–CF_3_ in DMSO, and subsequently, the reaction solution was added to PBS for measuring the spectral properties (Figure 2A).

In PBS (10 mM, pH = 7.4, 30% DMSO as co-solvent) solution, CT–CF_3_ exhibited an absorption maximum at 380 nm and was non-fluorescent. However, after reacting with O_2_^•–^, the fluorescence of CT–CF_3_ increased dramatically at 665 nm, while the absorption wavelength was red-shifted to 500 nm (Figure 2B,C). This change confirms that the reaction of CT–CF_3_ with O_2_^•–^ resulted in the release of CT-O^–^. As a strong electron-withdrawing group, the trifluoromethanesulfonyl group weakens the electron-donating ability of the phenolic anion, and the enhanced energy gap between the HOMO and LUMO results in a decrease of intramolecular charge transfer (ICT), which ultimately quenches the fluorescence of the CT–OH (Figure 2D). Whilst when O_2_^•–^ reacts selectively with triflate, recovery of ICT results in an enhancement of fluorescence. The reaction products of CT–CF_3_ (5 μM) and O_2_^•–^ (100 μM) were identified by high-performance liquid chromatography (HPLC) and high-resolution mass spectroscopy (HRMS) to confirm the proposed sensing mechanism (Figure S5). The retention time of CT–CF_3_ was 9.24 min, and the *m*/*z* was 457.0642 (Figure S5A,B). The retention time of the main reaction product (CT–OH) was 8.12 min, and the *m*/*z* was 325.1152 (Figure S5C,D). This result confirms that the nucleophilic reaction between CT–CF_3_ and O_2_^•–^ releases the fluorophore CT–OH.

When CT–CF_3_ reacted with O_2_^•–^ (17 μM), the fluorescence intensity increased almost 10 times (Figure 2C). Based on a linear relationship between the fluorescence intensity and O_2_^•–^ concentration, the limit of detection LOD = (3σ/slope) value for CT–CF_3_ was determined to be 0.079 μM (Figure S4A). This result indicates that CT–CF_3_ is highly sensitive toward O_2_^•–^. Given the potential interference of pH values, we determined the sensing performance of CT–CF_3_ for O_2_^•–^ in different pH environments. As shown in Figure S4B, the fluorescence intensity of CT–CF_3_ plateaued at pH = 7–8, which was in line with the physiological working pH *in vivo*. Furthermore, we evaluated the time course of the reaction of CT–CF_3_ with O_2_^•–^ (Figure S4C), and the fluorescence intensity stabilized after 7 min (includes 5 min of reaction with O_2_^•–^ in DMSO and 2 min to reach a plateau in aqueous solution). Given that the complex components of the physiological environment could affect the sensing signals, we evaluated the specificity of CT–CF_3_ (Figure S4D). Various metal ions (Na^+^, Fe^3+^, Cu^2+^, Fe^2+^, and Zn^2+^), ROS and RNS species (H_2_O_2_, ·OH, ^1^O_2_, O_2_^•–^., ClO^–^, ONOO^–^), representative reducing substances (HS^–^, GSH, Hcy, Cys), and bovine serum albumin (BSA) were evaluated to verify the exclusive reactivity of CT–CF_3_ toward O_2_^•–^. The interfering species exhibited negligible fluorescence changes when compared to O_2_^•–^. Therefore, CT–CF_3_ exhibits excellent selectivity for O_2_^•–^.

### Cytotoxicity and Hemolysis Rate

In order to use CT–CF_3_ for the staining of live cells and *in vivo* imaging, we evaluated the cytotoxicity and hemolysis rate of the CT–CF_3_. Cytotoxicity was evaluated by the MTT method.

The incubation of CT–CF_3_ at different concentrations (0, 2, 5, 10, 15, and 20 μM) with PC-12 cells for 24 h resulted in a survival rate of greater than 90% (Figure S4E). The hemolysis rate of CT–CF_3_ did not exceed 1% at a concentration of 200 μM, which was attributed to the good lipid solubility and weak hydrogen bonding ability of CT–CF_3_ (Figure S4F). The photostability of the probes was evaluated by continuous laser irradiation of CT–OH (10 μM) and compared with the commercial mitochondrial dye rhodamine 123 (5 μM) in A549 cells for 300 s (Figure S8). After 300 s of continuous laser irradiation, the average fluorescence intensity of rhodamine 123 was reduced by approximately 70%, while the average fluorescence intensity of the fluorophore CT–OH was reduced by approximately 30%. CT–OH has better photostability and can be used for cellular and in vivo imaging.

### Detection of O_2_^•–^ in Cells

**Figure 3:**
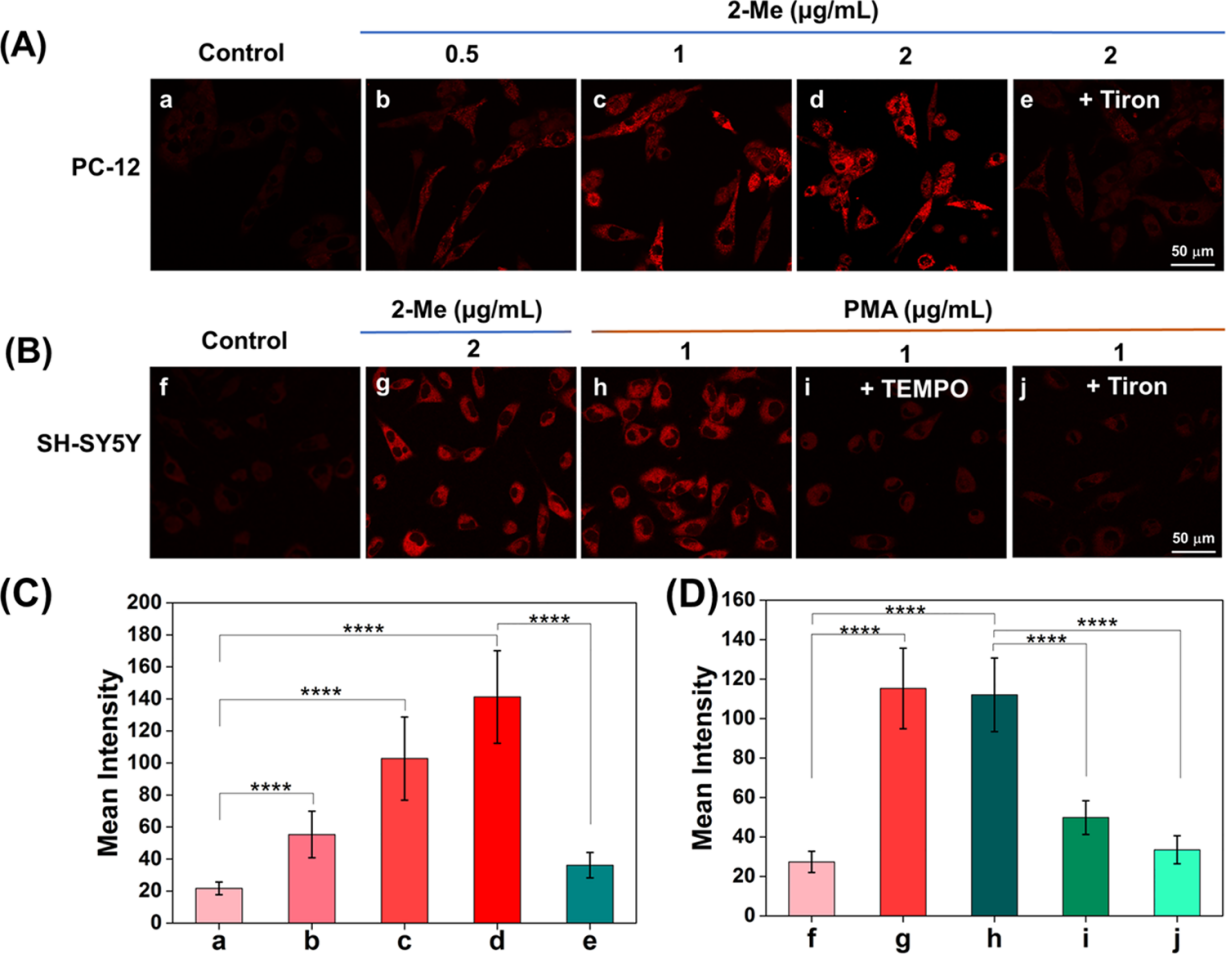
Imaging of intracellular O_2_^•–^. (A) Confocal fluorescence imaging of endogenous O_2_^•–^ in PC12 cells. 10 μM CT–CF_3_ loaded cells were co-incubated with 0, 0.5, 1, 2 μg/mL2-Me, and 2 μg/mL 2-Me + 200 μM Tiron for 30 min, respectively; (B) confocal fluorescence imaging of endogenous O_2_^•–^ in SH-SY5Y cells. (f) SH-SY5Y cells were incubated with 10 μM CT–CF_3_ and then imaged. (g) SH-SY5Y cells were pretreated with 2 μg/mL2-Me for 30 min, then incubated with 10 μM CT–CF_3_ for 90 min for imaging. (h) SH-SY5Y cells were pretreated with 1 μg/mLPMA for 30 min, then incubated with 10 μM CT–CF_3_ for 90 min for imaging. (i–j) SH-SY5Ycells were incubated with 1.0 μg/mL PMA for 0.5 h, then incubated with TEMPO (100 μM) and/or Tiron (200 μM) for 0.5 h, and finally incubated with 10 μM CT–CF_3_ for imaging. (C, D) Mean intensities in (A) (a–e) and (B) (f–j). λ_ex_ = 488 nm, λ_em_ = 630–720 nm. Scale bar = 50 μm. Data are presented as the mean value (*n* = 3), and the error bars were ± standard deviation (SD). ****P ≤ 0.0001.

To evaluate the sensing behavior of CT–CF_3_ in living cells, we used CT–CF_3_ to detect endogenous O_2_^•–^ in two types of neural cells (PC-12 cells and SH-SY5Y cells). PC-12 cells were pretreated with different doses of 2-methoxyestradiol (2-Me), a copper-zinc-manganese superoxide dismutase inhibitor, to increase the endogenous level of O_2_^•–^. As shown in Figure 3A, the intracellular fluorescence intensity of PC-12 was positively correlated with the dose of 2-Me (0, 0.5, 1, 2 μg/mL), which indicated that CT–CF_3_ could sense the enhancement of endogenous O_2_^•–^ in cells. Furthermore, Tiron (a superoxide scavenger) was added to the cells after pretreatment with 2-Me. There was a significant reduction of fluorescence compared to the group treated with 2-Me alone (Figure 3C). These results indicated that CT–CF_3_ could sensitively reflect the burst of endogenous O_2_^•–^ in PC-12 cells. Subsequently, CT–CF_3_ was used to sense oxidative stress in SH-SY5Y cells. Bright fluorescence was observed by co-culturing CT–CF_3_ with 2-Me-pretreated cells. Therefore, CT–CF_3_ could also be used for O_2_^•–^ sensing in SH-SY5Y cells (Figure 3B). Phorbol-12-myristate-13-acetate (PMA) was then used as a stimulator to trigger oxidative stress in cells. Obvious fluorescence was observed in PMA-treated cells compared to the control group, indicating increased intracellular O_2_^•–^ levels (Figure 3B). Morerover, two superoxide scavengers, 2,2,6,6-tetramethylpiperidine-N-oxyl (TEMPO) and Tiron, reduced O_2_^•–^ levels in PMA-treated cells, resulting in little fluorescent changes of CT–CF_3_ in the cells. Indicating that CT–CF_3_ could be used to monitor the changes of O_2_^•–^ when oxidative stress occurred in SH-SY5Y cells (Figure 3D).

Microglia are the most common immune cells in the central nervous system (CNS), as well as an important source of ROS in the brain.^34^ When inflammation, infection, trauma, and other neurological diseases occur in the brain, microglia are rapidly activated to form reactive microglia, which secrete high levels of inflammatory cytokines and ROS.^35,36^

**Figure 4:**
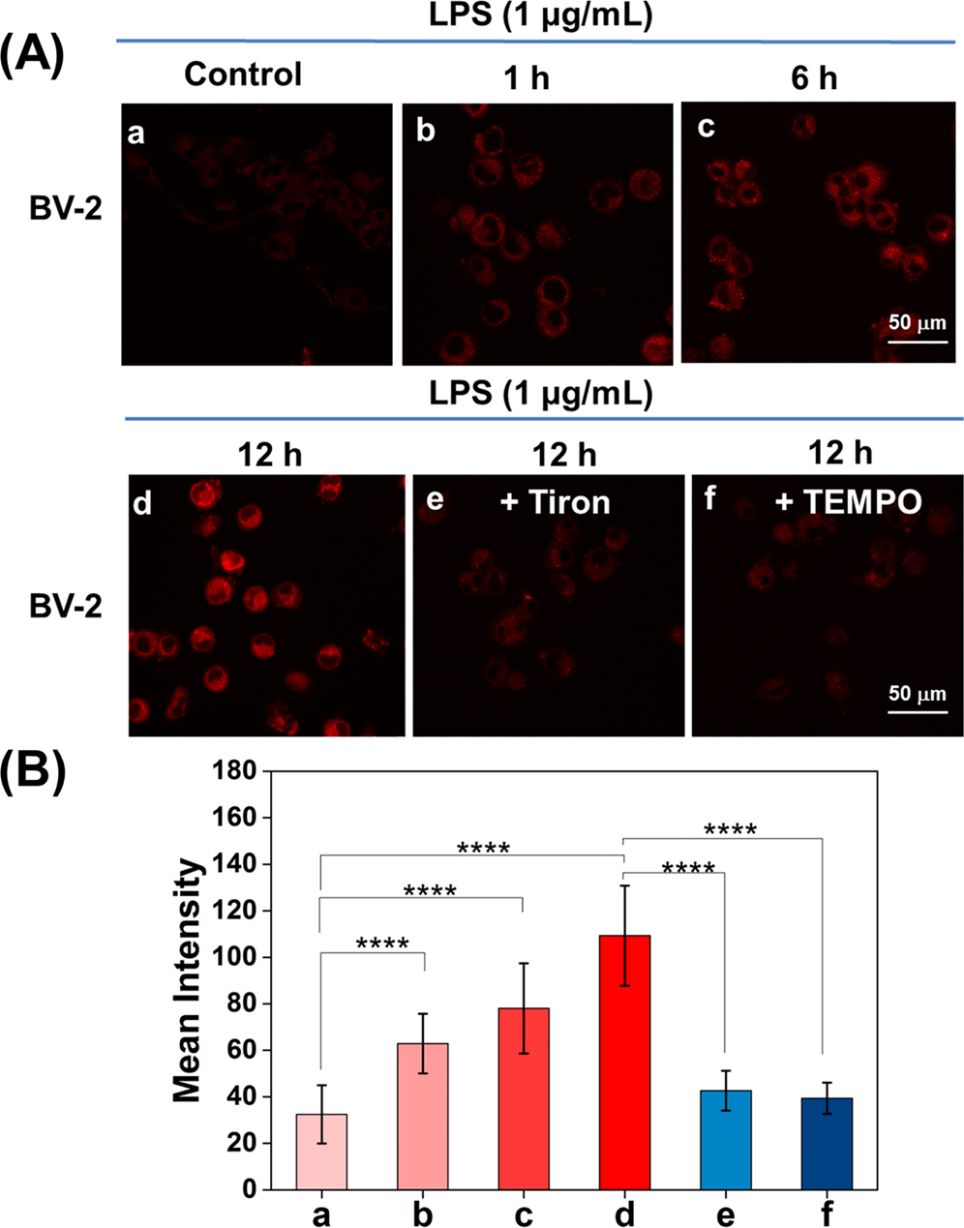
Monitoring of O_2_^•–^ in a cellular model of neuroinflammation. (A) Confocal fluorescence imaging of O_2_^•–^ produced by the LPS-induced oxidative stress in BV-2 cells. 10 μM CT–CF_3_ loaded cells were co-incubated with LPS (1 μg/mL) for (a) 0 h; (b) 1 h; (c) 6 h; (d) 12 h; (e, f) 10 μM CT–CF_3_ loaded cells were co-incubated with LPS (1 μg/mL) for 12 h, and (e) Tiron (200 μM); (f) TEMPO (100 μM) for 0.5 h. (B) Mean intensities in (A) (a–f); λ_ex_ = 488 nm, λ_em_ = 630–720 nm. Scale bar = 50 μm. Data were presented as the mean value (*n* = 3), and the error bars were ± standard deviation (SD). ****P ≤ 0.0001.

We used LPS-activated microglia to mimic neuroinflammation and oxidative stress in the brain. As shown in Figure 4, the intracellular fluorescence gradually intensified with increasing time of co-incubation with LPS (0, 1, 6, 12 h). LPS induced the activation of BV-2 cells and generated high levels of O_2_^•–^. After treatment of LPS-activated BV-2 cells with Tiron or TEMPO, CT–CF_3_ showed little enhancement of intracellular fluorescence intensity. This result indicates that high levels of intracellular O_2_^•–^ were reduced by both superoxide scavengers (Figure 4A,B). As such, the fluorescence of CT–CF_3_ was able to reflect the O_2_^•–^ fluctuations in a neuroinflammatory cellular model.

### Detection of O_2_^•–^ in the Brains of Neuroinflammatory Mice

Fluorescent probes for intracerebral O_2_^•–^ imaging require serum stability and excellent BBB permeability. CT–CF_3_ remained stable in serum in 3 h (hydrolysis rate < 10%) with a blood half-life of *t*_1/2_ = 0.88 ± 0.09 h, indicating that CT–CF_3_ reached the peak in 1 h and could be metabolized in 3–4 h (Figures S6, S7, and Table S2). In addition, CT–CF_3_ exhibits good lipid solubility (*c *Log *P*: 4.82), low molecular weight (Mol: 457), and weak hydrogen bonding ability (no hydroxyl, amine, carboxyl, and other groups). These properties endow CT–CF_3_ with excellent BBB permeability. We determined the absolute BBB permeability of CT–CF_3_ by HPLC (Figure S9). CT–CF_3_ was well absorbed by the brain with a brain uptake rate of 2.52% ID/g at 5 min. Therefore, CT–CF_3_ displays great potential for in vivo brain imaging.

**Figure 5:**
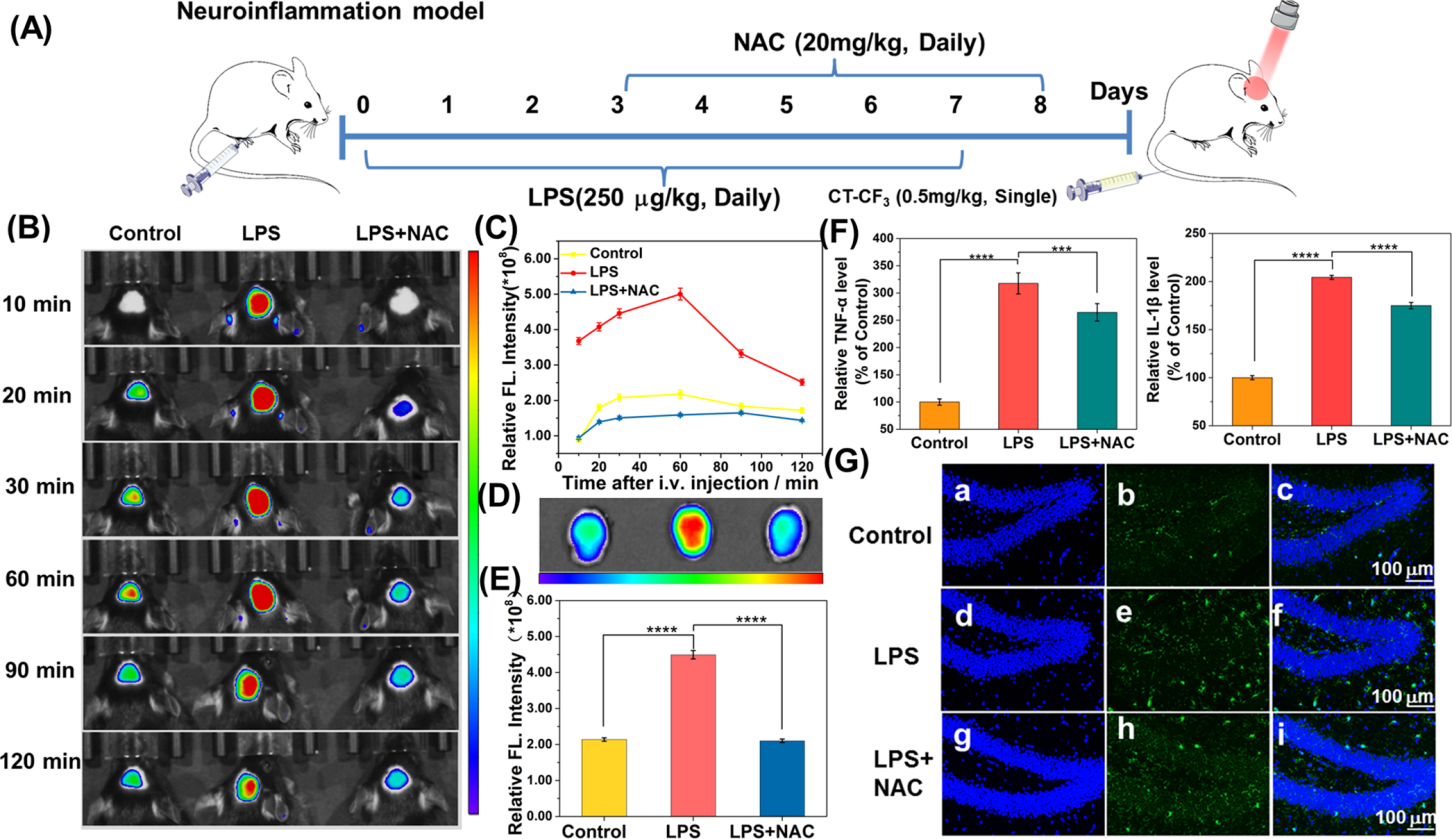
(A) Schematic diagram of the experimental design illustrating the duration of the lipopolysaccharide (LPS) and/or NAC administration in adolescent mice. (B) Mapping O_2_^•–^ fluxes in the brains of live mice with CT–CF_3_. Images were recorded after the intravenous (i.v.) injection of CT–CF_3_ at 10, 20, 30, 60, 90, and 120 min. (C) Curve of the fluorescence intensity of three mouse brains over time. (D) NIR Fluorescence images of isolated brains of the three mice. (E) Quantification of the fluorescence intensity of images in (D). (F) Effects of LPS and NAC on pro-inflammatory cytokine (TNF-α and IL-1β) levels in the brain. (G) Representative immunofluorescence photographs of microglia in the dentate gyrus of the hippocampus. Iba-1-positive microglia (green) and DAPI-granule cells (blue). Data are represented as the mean ± SD (*n* = 3), ****P ≤ 0.0001, ***P < 0.001.

Neuroinflammatory mice are modeled by intraperitoneal injection of LPS (Figure 5A).^24^ The neuroinflammation in the mice model was confirmed by increased cytokine levels of the tumor necrosis factor-α (TNF-α) and interleukin-1 β (IL-1β) of the brain tissue. Compared with the control group, the levels of TNF-α and IL-1β in the experimental group (LPS) increased by 3.2 times and 2 times, and the treatment group (LPS + NAC) increased by 2.6 times and 1.7 times (Figure 5F). We then injected three groups of mice with CT–CF_3_ (0.5 mg/kg) via the tail vein, and the fluorescence imaging of the brain was performed at different times up to 120 min. As shown in Figure 5B,C, the time-dependent fluorescent intensity was consistent with the levels of O_2_^•–^ in the brains of mice. On the basis of the successful uptake of CT–CF_3_ by the brain, the fluorescence intensity both in the control and experimental groups gradually increased and reached a plateau after around 60 min, then the brain clearance mechanism gradually dominated, resulting in a gradual decrease of the fluorescence. Notably, the bright fluorescence of the experimental group (LPS) was maintained throughout the imaging process and was almost 3 times stronger than that of the control group (Figure 5B,C). NAC could scavenge ROS of the brains,^37−39^ so the treatment group (LPS + NAC) exhibited only weak fluorescence. The fluorescence intensity was close to that of the control. The isolated brain tissues indicated that the fluorescence intensity of the experimental group (LPS) was significantly increased, while the treatment and control groups were similar (Figure 5D,E). The other isolated organs indicated that the fluorescence intensity of the experimental group (LPS) mouse kidney was significantly higher than the treatment group and the control group (Figure S11).

To understand the cause and damage of high levels of O_2_^•–^ expression in the neuroinflammatory brain, we performed immunofluorescence staining and hematoxylin and eosin (H&E) staining on organ tissue sections of mice. Immunolabeling Iba-1 was used to detect microglial activation of the brain (Figures 5G and S12). The control group displayed a few green Iba-1 positive resting microglia, which have a small and compact morphology.

While the experimental group (LPS) displayed a significant increase in the number of activation-positive microglia and changes of cell morphology, including cell hypertrophy, increased retraction, and decreased branching. On the other hand, the treatment group (LPS + NAC) displayed a reduction in the number of positive microglia and morphological changes. H&E staining indicated that prolonged activation of the microglia could damage neurons in the hippocampus (Figure S13). In the experimental group, the neuronal necrosis and neurophagy in the CA1-3 area of the brain tissue increased and the nuclear pyknosis in the dentate gyrus (DG) of the hippocampus increased. The neurons in the control group were closely arranged, and the nuclei were lightly stained and clear. There was no obvious damage to the neurons in the CA1-3 area, but a small amount of neuron pyknosis and necrosis appeared in the DG area. These results provide substantial biological evidence for the existence of oxidative stress in the brain of neuroinflammatory mice. In addition, due to the long-term stimulation by LPS, the liver of the mice in the experimental group displayed obvious redness and swelling, and the spleen was abnormally enlarged (Figure S10A). H&E staining indicated enhanced congestion and hemorrhage spots in the liver tissue of the experimental mice (Figure S14). Based on the results from *in vivo* fluorescence imaging using CT–CF_3_, immunofluorescence staining, pro-inflammatory cytokine detection, and H&E staining, LPS-activated microglia resulted in neuroinflammation and promoted bursts in high levels of O_2_^•–^, which subsequently resulted in the oxidative damage of neurons. Significantly, CT–CF_3_ is the first fluorescent probe capable of monitoring O_2_^•–^ fluctuations in the brain of neuroinflammatory mice.

### Detection of O_2_^•–^ in the Brains of SZ Mice

**Figure 6:**
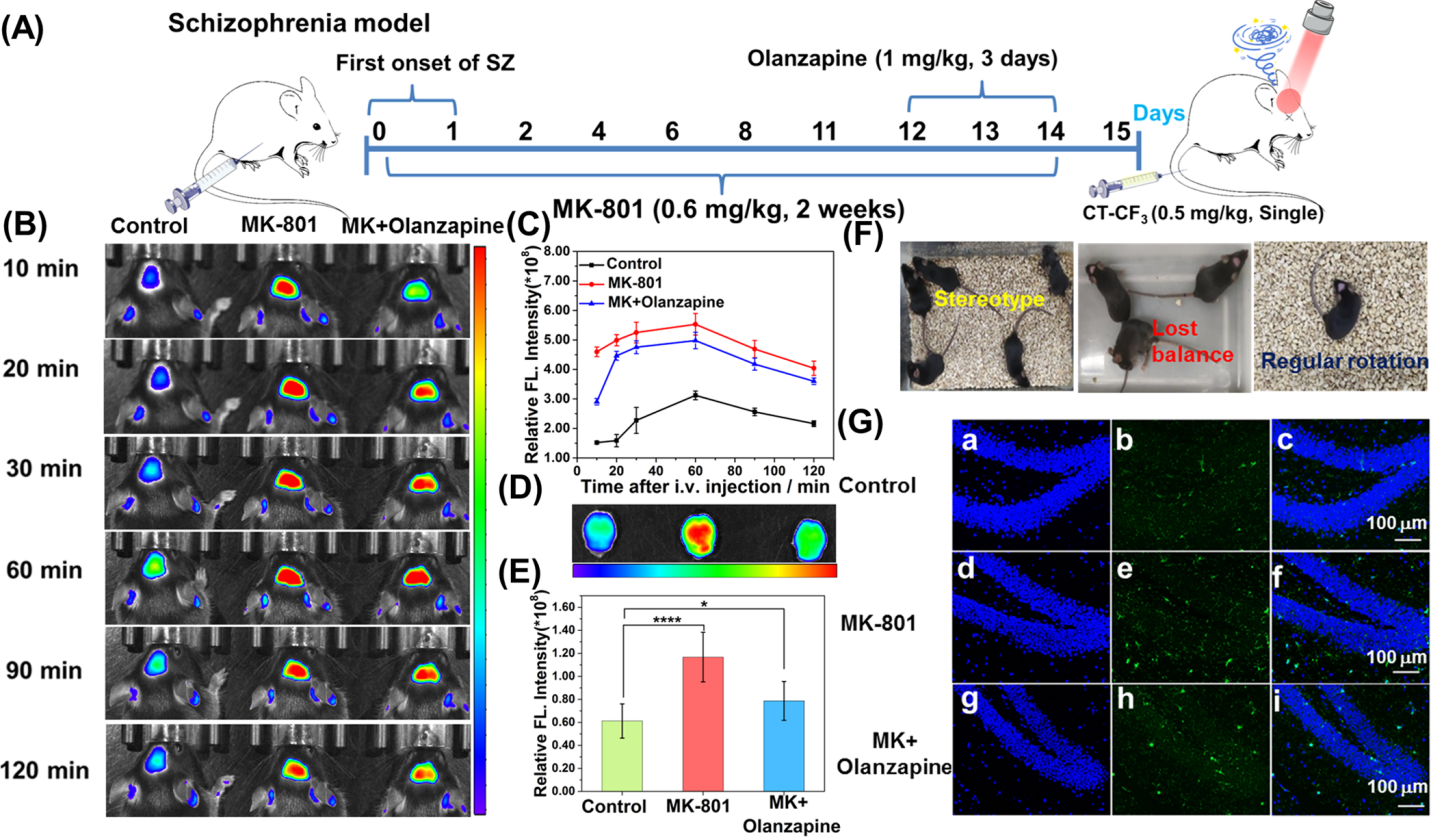
(A) Schematic diagram of the experimental design showing the duration of the Dizocilpine (MK-801) and/or Olanzapine administration in adolescent mice. (B) Mapping O_2_^•–^ fluxes in the brains of live mice with CT–CF_3_. Images were recorded after the intravenous (i.v.) injection of CT–CF_3_ at 10, 20, 30, 60, 90, and 120 min. (C) Curve of the fluorescence intensity of three mouse brains over time. (D) NIR Fluorescence images of isolated brains of the three mice. (E) Quantification of the fluorescence intensity of images in (D). (F) Screenshots of some abnormal behaviors (including stereotyped and ataxia behavior) in schizophrenia mice are from the Supporting Information Movie 1–3. (G) Representative immunofluorescence photographs of microglia in the DG region of the hippocampus. Iba-1-positive microglia (green) and DAPI-granule cells (blue). Data are represented as the mean ± SD (*n* = 3), ****P ≤ 0.0001, ***P < 0.001.

Due to the sensitivity and specificity of CT–CF_3_ to O_2_^•–^*in vitro* and *in vivo*, we were encouraged to explore the relationship between oxidative stress and the pathology of schizophrenia. As shown in Figure 6A, we established a SZ mouse model by the long-term injection of a low dose of Dizocilpine (MK-801) (14 days, 0.6 mg/kg) and evaluated the behavior following protocols from previous research.^40,41^ Some specific behaviors of SZ mice (such as head wiggling or twitching, regular rotation, unsteady walking, and falling sideways) are recorded in movies (Figure 6F and Supporting Information Movies 1 and 2). MK-801 as a N-methyl-d-aspartate receptor (NMDAR) antagonist is widely used to induce schizophrenia in mice.

After the three groups of mice were injected with CT–CF_3_ (0.5 mg/kg) via the tail vein, fluorescence imaging in the brains of mice was performed for 120 min (Figure 6B,C). Over the first 60 min, the brain uptake rate of the probe was greater than the brain clearance rate, CT–CF_3_ reacted with high levels of O_2_^•–^ to generate an enhancement of fluorescence in the mouse brain (Figure 6B,C). Subsequently, the clearance mechanisms of the brain gradually predominated, resulting in a gradual decrease of fluorescence in the brain. During the course of imaging, the brains of mice in the experimental group (MK-801) exhibited bright fluorescence, which was almost 2–3 times stronger than the control group (Figure 6B,C). Olanzapine, atypical antipsychotics with antioxidant properties, was used as a therapeutic drug (1 mg/kg, 3 days).

Compared with the experimental group, the fluorescence intensity of the treatment group decreased but was still 1.5–2 times higher than that of the control group. The fluorescence intensity of the isolated brain tissue was consistent with that of *in vivo* imaging (Figure 6D,E). The experimental group displayed a 1.9-fold increase in fluorescence intensity, whilst the treatment group displayed a 1.28-fold increase (Figure 6E). Other isolated organs confirmed that the fluorescence intensity of the metabolic organs (kidney and liver) in the experimental group and the treatment group was significantly higher than the control group (Figure S15). To understand the etiology and neuronal changes of high O_2_^•–^ expression in the brains of SZ mice, we performed immunofluorescence staining, and hematoxylin and eosin (H&E) staining on organ tissue sections. Immunolabeling Iba-1 was used to detect microglial activation in the brain tissue (Figures 6G and S16). In the hippocampus and cortex, a small number of Iba-1-positive (green) resting microglia were shown in the control group, and the number of activated-positive microglia was significantly increased in the experimental and treatment groups. The results of H&E staining confirmed the damage to neurons in the hippocampus by prolonged activation of the microglia (Figure S17). Compared with the control group, the neurons in the hippocampus of the experimental group exhibited pyknosis, atrophy, and necrosis damage, and the neurons in the CA3 area were disordered. Compared with the experimental group, the neuronal necrosis and atrophy in the treatment group were improved, but the neurons in the CA3 area were also disordered. In addition, H&E staining of other organs indicated no severe lesions (Figure S18), but the liver and kidney damage of the treated mice was increased, which may be attributed to the increased organ burden with the combined use of two drugs.

The fluorescence imaging of CT–CF_3_*in vivo*, immunofluorescence staining and H&E staining provided evidence that prolonged activation of microglia in the brains of SZ mice produces high levels of O_2_^•–^, which could result in neuronal damage.^42^ The short-term treatment with atypical antipsychotic drugs can improve oxidative stress and neuronal damage. In order to obtain a better therapeutic effect, long-term use of drugs and adjuvant therapy with antioxidants may be required.

Early diagnosis and effective intervention (e.g., initial selection and use of multiple antipsychotics) are critical to achieving long-term positive clinical outcomes for the first episode of SZ.^43^ We established a mouse model for the first SZ episode using a single injection of MK-801 into adolescent mice (C57/BL6, 6 weeks). As shown in Figure S19A,B, the brains of four mice were subjected to fluorescence imaging at 60 min after CT–CF_3_ injection. Compared with the control group, the fluorescence intensity in the brains in the experimental group (MK-801) increased by 2 times, and the fluorescence intensity in the brains treated with olanzapine and risperidone was increased by 1.5 times and 1.6 times, respectively (Figure S19B). The fluorescence intensity of the isolated brain tissue was consistent with *in vivo* imaging (Figure S19C), but the gap between the fluorescence intensity of the control group and the other three groups was larger 2–3 times (Figure S19D). These results confirmed that oxidative stress in the brains of MK-801-induced SZ mice was not the result of a long-term effect, and the first stimulation could lead to a burst of O_2_^•–^. Furthermore, a single treatment with two atypical antipsychotic drugs was not effective in suppressing oxidative stress in the brain.

## Conclusions

With this research, we developed an O_2_^•–^ activated NIR probe CT–CF_3_ in order to evaluate the relationship between oxidative stress, neuroinflammation, and schizophrenia. From our research, the monitoring of oxidative stress can facilitate the diagnosis and enable an assessment of schizophrenia progression. CT–CF_3_ exhibits high sensitivity, good selectivity, and biocompatibility for O_2_^•–^. At the cellular level, CT–CF_3_ was successfully used to monitor the O_2_^•–^ concentration changes in nerve cells and microglia undergoing oxidative stress. On the basis of the excellent BBB penetration ability of CT–CF_3_, we could visualize the dynamic changes in O_2_^•–^ flux in neuroinflammatory and SZ mouse brains for the first time. The immunofluorescence and H&E staining of brain slices confirmed prolonged activation of microglia and neuronal damage, providing biological evidence for oxidative stress in the brains of neuroinflamed mice and SZ mice. With the aid of CT–CF_3_, a burst of O_2_^•–^ in the brain of mice with the first episode of SZ was confirmed, and the effect of two atypical antipsychotics (risperidone and olanzapine) on redox homeostasis was assessed in the brain. We envision this work will expand the application of fluorescent probes for understanding the chemical processes of the brain and could help evaluate the link between oxidative stress and a variety of brain diseases.

## Supporting Information Available

Supplementary data, characterization figures of compounds (PDF)
Abnormal behavior in schizophrenic mice (Movie 1) (MP4)
Specific behavior of schizophrenic mice (Movie 2) (MP4)
Schizophrenic mouse abnormal behavior (Movie 3) (MP4)
