# Supplementary material for: Near-Infrared Fluorescent Probe for the In Situ Visualization of Oxidative Stress in the Brains of Neuroinflammatory and Schizophrenic Mice
Source: Anal Chem. 2023 Aug 1;95(32):11943–52. doi: 10.1021/acs.analchem.3c01447 (PMC10433243; doi:10.1021/acs.analchem.3c01447)
Supplement: Supplementary file 1 — ac3c01447_si_001.pdf [file ac3c01447_si_001.pdf]

Supporting Information

A near-infrared fluorescent probe for the In situ visualization of oxidative stress in the brains of neuroinflammatory and schizophrenic mice

Yujie Geng, Hanchen Zhang, Guoyang Zhang, Jiaying Zhou, Mingguang Zhu, Lijun Ma, Xuefei Wang, Tony D. James* and Zhuo Wang**

Y. Geng, G. Zhang, J. Zhou, M. Zhu, L. Ma, Prof. Z. Wang
State Key Laboratory of Chemical Resource Engineering, College of Chemistry,
Beijing Advanced Innovation Center for Soft Matter Science and Engineering,
Beijing University of Chemical Technology, Beijing, 100029, China.
E-mail: wangzhuo77@mail.buct.edu.cn

Prof. T. D. James
Department of Chemistry, University of Bath, BA2 7AY, UK and the
School of Chemistry and Chemical Engineering, Henan Normal University, Xinxiang 453007, China.
Email: T.D.James@bath.ac.uk

Prof. X. Wang
School of Chemical Sciences
University of Chinese Academy of Sciences
Beijing, 100049, China.
E-mail: wangxf@ucas.ac.cn

H. Zhang
Institute of Chemistry, Chinese Academy of Sciences Zhongguancun North First Street 2,100190 Beijing, PR China

Table of Contents.

Experimental Section S2
Materials and instruments S2
General Procedure for Fluorescence Detection S2
In vitro cytotoxicity assay S2
Hemolysis experiment S3
The BBB Permeability of CT-CF_3 .... S3
Prediction of the molecular lipid water partition coefficient (*c* Log *P*) S4
Preparation of LPS-Induced neuroinflammation mouse model S4
Detection of pro-inflammatory cytokines S4
Preparation of schizophrenia mouse model and fluorescence imaging S5
Paraffin Section Immunofluorescence S5
Behavior analysis S7
Synthesis and characterization S8-S9
Table S1.The spectral data of Fluorophores S10
Figure S4 The spectral data of CT-CF_3 .... S11
Figure S5 HPLC profiles of CT-CF_3 and CT-CF_3+KO_2. .... S11
Table S2 Pharmacokinetic parameters of CT-CF_3 in plasma S12
Figure S7-S8 Serum stability and photostability studies of CT-CF_3 .... S13
Figure S9 Determination of the absolute BBB permeability of CT-CF_3 by HPLC S14
Figure S10 Organ and body weight alterations in neuroinflamed mice. S14
Figure S11 Imaging of isolated organs in neuroinflamed mice. S15
Figure S12 The representative immunofluorescence photographs of microglia in the cortex region of neuroinflammation mice S15
Figure S13 H&E staining for evaluating neuronal damage in the hippocampal region . S16
Figure S14 H&E staining of major organs of neuroinflammation mice. S16
Figure S15 NIR Fluorescence images of isolated organs of the three mice S17
Figure S16 The representative immunofluorescence photographs of microglia in the cortex region of SZ mice S17
Figure S17 H&E staining for evaluating neuronal damage in the hippocampal region . S18
Figure S18 H&E staining of major organs from SZ mice. S18
Figure S19 Mapping $O_2^{\bullet-}$ fluxes in the brains of mice with first-onset schizophrenia.... S19
Figure S20. ^{1}H NMR spectrum of CN-OH S20
Figure S21. ^{1}H NMR spectrum of CF-OH S20
Figure S22. ^{1}H NMR spectrum of CT-OH S21
Figure S23. ^{13}C NMR spectrum of CT-OH S21
Figure S24. ^{1}H NMR spectrum of CT-CF_3 .... S22
Figure S25. ^{13}C NMR spectrum of CT-CF_3 .... S22
Figure S26. MS spectrum of CN-OH S23
Figure S27. MS spectrum of CF-OH S23
Figure S28. HRMS spectrum of CT-OH S24
Figure S29. HRMS spectrum of CT-CF_3 .... S25
References S26

Experimental Section

Materials and instruments

All raw materials are analytical grade and are used directly unless otherwise stated. All ethanol in the synthesis step are further dried. Schizophrenia stimulating drugs MK-801(+) was purchased from Bide Pharmatech Ltd (China). $O_2^{\bullet-}$ was produced from KO_2 in dry DMSO by an ultrasonic method. The concentration of $O_2^{\bullet-}$ was determined from the absorption at 250 nm (ε = 2682 M^{-1} cm^{-1}). The preparation of reactive oxygen species and reactive nitrogen species refers to the methods reported previously. [1]

UV–vis absorption and fluorescence spectra were obtained on HITACHI U-3900H and HITACHI F-4600 spectrophotometers, respectively. The cells used were purchased from China Cell Bank. The MTT cytotoxicity test kit was purchased from Solarbio. In vitro imaging was carried out using a laser scanning confocal microscope (Leica SP5). *In vivo* imaging was conducted using an IVIS Spectrum Imaging System.

General Procedure for Fluorescence Detection

We prepared different concentrations of $O_2^{\bullet-}$ and CT-CF_3 in DMSO solutions (300 μL). After 5 min of preparation, diluted the mixture to 1 mL with PBS buffer (10 mM, pH 7.4) and incubated for 5 min before measurement. The final concentration of CT-CF_3 in PBS buffer solution (30% DMSO) was 5 μM. $\lambda_{ex/em}$ = 500/670 nm.

In vitro cytotoxicity assay

The cytotoxicity of the CT-CF_3 was tested by the MTT (methyl thiazolyl tetrazolium) method. In a humid environment of 37°C and 5% CO_2, PC-12 cells were incubated in high sugar Dulbecco's modified Eagle's medium (DMEM) containing 10% fetal bovine serum. Subsequently, the cells were collected and planted into 98-well plates. After 24 h of incubation, the original medium was removed, and the cells containing CT-CF_3 (0, 2, 5, 10, 15 and 20 μM) serum-free DMEM was added to the plate. After 24 h of co-incubation, 50 μL of MTT was added to each well to interact with live cells to produce metsan. After another 4 h incubation, the old medium was removed and 150 μL of dimethyl sulfoxide (DMSO) was added to each well and placed on a

shaking incubator for 10 min. Finally, the cell viability was determined by measuring the ultraviolet absorption value in different well plates by a microplate reader.

Hemolysis experiment

Take 1mL of mouse blood, add 1mL PBS and dilute to 2 mL. The sample was then centrifuged at 8000 rpm for 10 min. After removing the supernatant, add PBS again to 2 mL for 10 min (8000 rpm). Repeat this process 5 times to obtain blood cells from the serum. Subsequently, the blood cells were diluted into 10 mL PBS, and each time 0.2 mL of blood cell solution was taken out and mixed with 0.8 mL distilled water, PBS and 0.8 mL CT-CF_3 gradient solutions (10, 20, 50, 100, 150, 200 μM) let stand for 3 h at room temperature. Finally, centrifuge (12000 rpm, 5 min) and aspirate the supernatant, and tested its ultraviolet absorption (Abs) by a microplate reader. Use the following formula to calculate the hemolysis rate (HR) of red blood cells:

$$\mathbf{HR = (Abs_{sample} - Abs_{PBS}) / (Abs_{H2O} - Abs_{PBS})}$$

The BBB Permeability of CT-CF_3.

We determined the BBB penetration rate of CT-CF_3 by High-performance liquid chromatography (HPLC). The instrument for HPLC is Agilent.1260 infinity ll, and Poroshell 120 column (4 μm C18, 4.6 mm × 150). A soulution of CT-CF_3 (0.5 mg/kg, in 3:7 DMSO/PBS) was inject into C57BL/6J mice (n=3, 15-18 g, 7 weeks, female) through intravenous injection. The mice were sacrificed and dissected at 5 min. Brain samples (The weight of the brain = 0.42 g ± 0.2 g) were removed, weighted and homogenized with 1.0 mL acetonitrile and then the leftover homogenate was extracted with 1.0 mL acetonitrile for twice, the total volume of acetonitrile was 3.0 mL. The brain tissues of all three mice were extracted in the same way. The extracted acetonitrile was filtered by flashing nylon membrane (0.22 μm) to analyze by HPLC. We mapped the standard curve of CT-CF_3 by HPLC, and determined the C_{probe} by the standard curve. Quantitative analysis was derived from peak area and the brain uptake was presented by % injected dose per gram (% ID/g). The calculation formula was shown below.

$$Uptake = \frac{m_{probe\_brain}}{\frac{m_{probe\_inject}}{m_{brain}}} \times 100\%$$

Where $\boldsymbol{m}_{\textbf{probe\_inject}}$ means the quality of injection. $\boldsymbol{m}_{\boldsymbol{brain}}$ means the wet weight of brain. $\boldsymbol{m}_{\boldsymbol{probe\_brain}}$ means the quality of probe in brain, which was calculated according to the fomula below.

$$m_{probe\_brain} = \mathrm{C}_{\mathrm{probe}} \bullet \mathrm{V}$$

Where C_{probe} was calculated by the peak area according to the standard curve, V means volume of extract (3 mL).

Prediction of the molecular lipid water partition coefficient (*c* Log *P*)

The *c* Log *P* values were calculated using the online ALOGPS 2.1 program.

Preparation of LPS-Induced neuroinflammation mouse model and fluorescence imaging

Mouse models of neuroinflammation were prepared according to previously reported methods. [2] 7-weeks-old male C57BL/6J mice (n =12) that were supplied by Beijing HFK BIOSCIENCE Co., Ltd. On the eighth day, one mouse was randomly selected from each of the three groups, and were simultaneously injected with CT-CF_3 (0.5 mg/kg, in 3:7 DMSO/PBS, v/v) through the tail vein, and then immediately placed in the IVIS Spectrum imaging system with an excitation filter of 500 nm and the collection wavelength range is from 640-720 nm.

After the imaging experiments were completed, the mice were sacrificed by cervical dislocation. Immediately dissect out the brain, heart, liver, spleen, lung, kidney and other tissues for fluorescence imaging. Subsequently, they were placed in a 10% formalin solution for paraffin embedding and tissue sectioning.

Detection of pro-inflammatory cytokines

Brain tissue sampling was performed one day after the end of administration. Three mice from different groups were randomly selected and sacrificed with CO_2, and the brain tissue was removed on an ice bath. Subsequently, the blood was quickly washed with PBS (4°C, pH = 7.4) solution and the surface water of the brain tissue was blotted dry and placed in a sterile 1.5 mL EP tube. Immediately, perform liquid

nitrogen irrigation, and then store in a -80°C refrigerator for later use. The above mouse brain tissue was homogenized in RIPA buffer, then centrifuged at 20000 rpm for 30 min at 4 °C, and the supernatant was collected. The levels of tumor necrosis factor-α (TNF-α) and interleukin-1 (IL-1β) were quantified with commercial ELISA kits (MULTI SCIENCES, China).

Paraffin Section Immunofluorescence

The paraffin section immunofluorescence experimental procedure is divided into 9 steps; i) Deparaffinize and rehydrate: incubate sections in 2 changes of xylene, 15 min each. Dehydrate in 2 changes of pure ethanol for 5 min, followed by dehydrate in gradient ethanol of 85% and 75% ethanol, respectively, 5 min each. Wash in distilled water; ii) Antigen retrieval: immerse the slides in EDTA antigen retrieval buffer (pH=8.0) and maintain at a sub-boiling temperature for 8 min, standing for 8 min and then followed by another sub-boiling temperature for 7 min. Be sure to prevent buffer solution evaporate. Let air cooling. Wash three times with PBS (pH=7.4) in a Rocker device, 5 min each. Use the right antigen retrieval buffer and heat extent according to tissue characteristics; iii) Circle and Serum blocking: eliminate obvious liquid, mark the objective tissue with liquid blocker pen. Add 3% BSA to cover the marked tissue to block non-specific binding for 30 min. Cover objective area with 10% donkey serum (for the case of primary antibody originated from goat) or 3% BSA (for the case of primary antibody originated from others); iv) Primary antibody: throw away the blocking solution slightly. Incubate slides with primary antibody (Iba-1: 1:500, Servicebio, China) overnight at 4°C, placed in a wet box containing a little water; v) Secondary antibody: wash slides three times with PBS (pH=7.4) in a Rocker device, 5 min each. Then throw away liquid slightly. Cover objective tissue with secondary antibody (Alexa Fluor 488: 1:400, Servicebio, China), incubate at room temperature for 50 min in dark condition; vi) DAPI counterstain in nucleus: wash three times with PBS (pH=7.4) in a Rocker device, 5 min each. Then incubate with DAPI solution at room temperature for 10 min, kept in dark place; vii) Spontaneous fluorescence quenching: wash three times with PBS (pH=7.4) in a Rocker device, 5 min each. Add spontaneous fluorescence quenching reagent to incubate for 5 min. Wash in running

tap water for 10 min. viii) Mount: Throw away liquid slightly, then cover slip with anti-fade mounting medium; ix) Microscopy detection and collect images by Fluorescent Microscopy.

Preparation of schizophrenia-first-episode mouse model and fluorescence imaging

Mouse models of schizophrenia were prepared according to previously reported methods. The nonecompetitive NMDA receptor antagonist dizocilpine hydrogen maleate (MK-801) was used to make a mouse model of schizophrenia, which was a widely used schizophrenia stimulating drug. [3] Low-dose and long-term repeated administration of MK-801 can induce long-lasting cognitive deficits and changes in brain nerve structure in adolescent or adult rats. [4] 6-weeks-old male C57BL/6J mice (n =12) that were supplied by Beijing HFK BIOSCIENCE Co., Ltd. On the fifteenth day, one mouse was randomly selected from each of the three groups, and were simultaneously injected with CT-CF_3 (0.5 mg/kg, in 3:7 DMSO/PBS, v/v) through the tail vein, and then immediately placed in the IVIS Spectrum imaging system with an excitation filter of 500 nm and the collection wavelength range is from 640-720 nm. After the imaging experiments were completed, the mice were sacrificed by cervical dislocation. Immediately, brain, heart, liver, spleen, lung, kidney and other tissues were dissected out for fluorescence imaging. Various types of tissues were placed in 10% formalin solution and further used for paraffin embedding and tissue sectioning.

We prepared mice of schizophrenia-first-episode with a single injection of MK-801 into healthy adolescent mice. 6-weeks-old male C57BL/6J mice (n =12) that were supplied by Beijing HFK BIOSCIENCE Co., Ltd., were randomly divided into four groups: i) the control vehicle (PBS) administered group; ii) the MK-801-treated (0.6 mg/kg, single) group; iii) the Olanzapine-treated (1 mg/kg, single) + MK-801-treated (0.6 mg/kg, single) group; iv) the Risperidone-treated (1 mg/kg, single) +MK-801-treated (0.6 mg/kg, single) group. After 60min of MK-801 injection, olanzapine or risperidone was injected by intraperitoneal injection. The injection time was between 14:00-15:00. After 6 h in all drug injections, one mouse was randomly selected from each of the four groups, and were simultaneously injected with CT-CF_3

(0.5 mg/kg, in 3:7 DMSO/PBS, v/v) through the tail vein, and then immediately placed in the IVIS Spectrum imaging system with an excitation filter of 500 nm and the collection wavelength range was 640-720 nm. After the imaging was completed, the mice were sacrificed by neck dissection and the brain tissue was rapidly dissected out for fluorescence imaging.

Behavior determination

About 20 min after intraperitoneal injection of MK-801, SZ mice showed obvious behavioral abnormalities. It was consistent with the previously reported behavior of schizophrenic mice prepared by MK-801 stimulation. [5] Generally, MK-801-induced rodent models of schizophrenia show three types of abnormal behaviors: i) Significant increase in the number of stereotyped behaviors, including head swinging or twitching, rotation, etc. (Supporting Movie 1); ii) Significant increase in the number of ataxia behaviors, including abduction of both hind limbs and unsteady walking, falling sideways, etc. (Supporting Movie 2); iii) Lack of response to sound and light. SZ mice exhibit prolonged (greater than 20 min) stereotyped rotations (clockwise, 5-15 cm in diameter) that cannot be interrupted by external forces (Supporting Movie 3). In addition, the mice in the treatment group behaved differently from the other mice, manifested by a significant reduction in the amount of exercise and prolonged standing time.

Synthesis and characterization

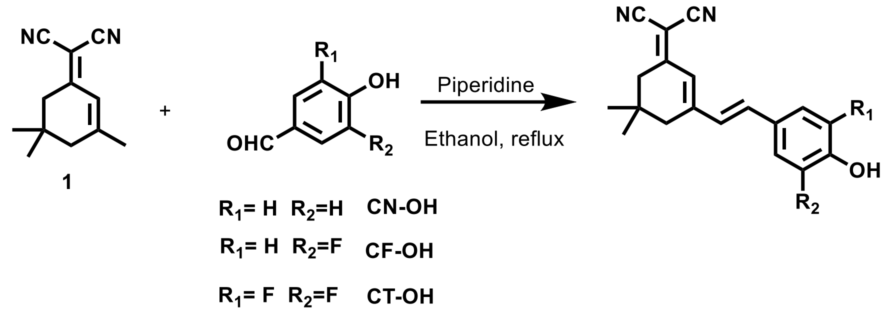

Figure S1 Synthesis of CN-OH, CF-OH and CT-OH.

1.1 Synthesis of CN-OH

Compound 1 (37.2 mg, 0.2 mmol) and p-hydroxybenzaldehyde (24.4 mg, 0.2 mmol) were added to a solution of ethanol (10 mL) containing catalytic amount of piperidine and refluxed under N_2 atmosphere for 6 h. Then, the mixture was evaporated under reduced pressure. The residue was purified by column chromatography (petroleum ether: CH_2Cl_2=1:1) to obtain an orange solid (43.7 mg, 75.4%). ^{1}H NMR (400 MHz, DMSO-d_6) δ 9.95 (s, 1H), 7.55 (s, 2H), 7.22 (d, J = 4.7 Hz, 2H), 6.84 – 6.76 (m, 3H), 2.61 (s, 2H), 2.54 (s, 2H), 1.02 (s, 6H). MS (MALDI-TOF-MS) m/z: cacld for $C_{19}H_{18}N_2O$, 291.14; found 291.23.

1.2 Synthesis of CF-OH

Compound 1 (37.2 mg, 0.2 mmol) and 3-Fluoro-4-hydroxybenzaldehyde (28 mg, 0.2 mmol) were added to a solution of ethanol (10 mL) containing catalytic amount of piperidine and refluxed under N_2 atmosphere for 6 h. Then, the mixture was evaporated under reduced pressure. The residue was purified by column chromatography (petroleum ether: CH_2Cl_2=1:1) to obtain an orange solid (35.6mg, 57.8%). ^{1}H NMR (400 MHz, Methanol-d_4) δ 7.40 (dd, J = 12.4, 2.1 Hz, 1H), 7.28 (d, J = 6.5 Hz, 1H), 7.16 (d, J = 16.0 Hz, 1H), 7.03 (d, J = 16.1 Hz, 1H), 6.94 (t, J = 8.7 Hz, 1H), 6.84 (s, 1H), 2.64 (s, 2H), 2.56 (s, 2H), 1.10 (s, 6H). MS (MALDI-TOF-MS) m/z: cacld for $C_{19}H_{18}N_2O$, 309.14; found 309.22.

1.3 Synthesis of CT-CF_3

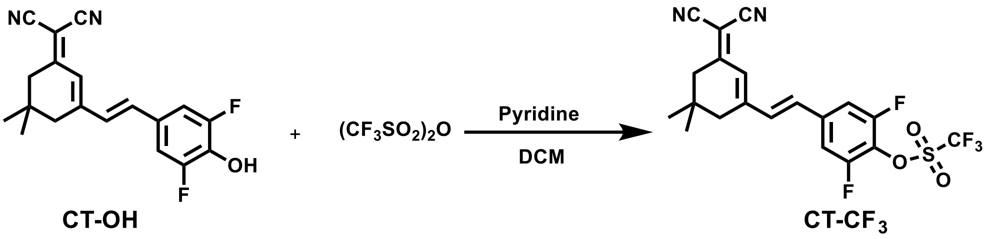

Figure S2 Synthesis of CT-CF_3.

Table S1 Summary of the spectral properties of the three fluorophores.

Compound	λ_{abs} (nm)	ξ^{a} ($\times10^{4}$)	λ_{em} (nm)	φ^{b}	$\triangle\lambda$ (nm)	pKa[c]
CN-OH	430	6.5	580	*	150	*
	500	0.01	663	0.0197	133	8.58
CF-OH	430	4.3	*	*	*	*
	500	2.0	665	0.0186	155	7.02
CT-OH	500	4.0	665	0.0177	165	5.68

a. Test results were obtained in PBS (7.4) solution. b. The test condition is 1M NaOH solution, with fluorescein as the reference. C. In a buffered solution containing 30% DMSO at pH 3-12. *represent no results available or not tested.

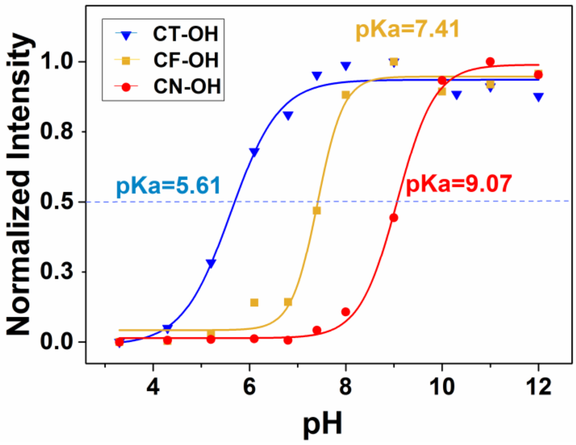

Figure S3 Plots of fluorescence intensity at 500 nm as a function of pH.

Absorption and fluorescence can be used for the study of the pKa of small molecule compounds (Figure S3). The pKa values were studied by testing the fluorescence intensity of three fluorophores (CN-OH, CF-OH and CT-OH) at 665 nm in different pH solutions, which were 9.07, 7.41 and 5.61 respectively. There was no significant difference in the pKa obtained using absorption or fluorescence.

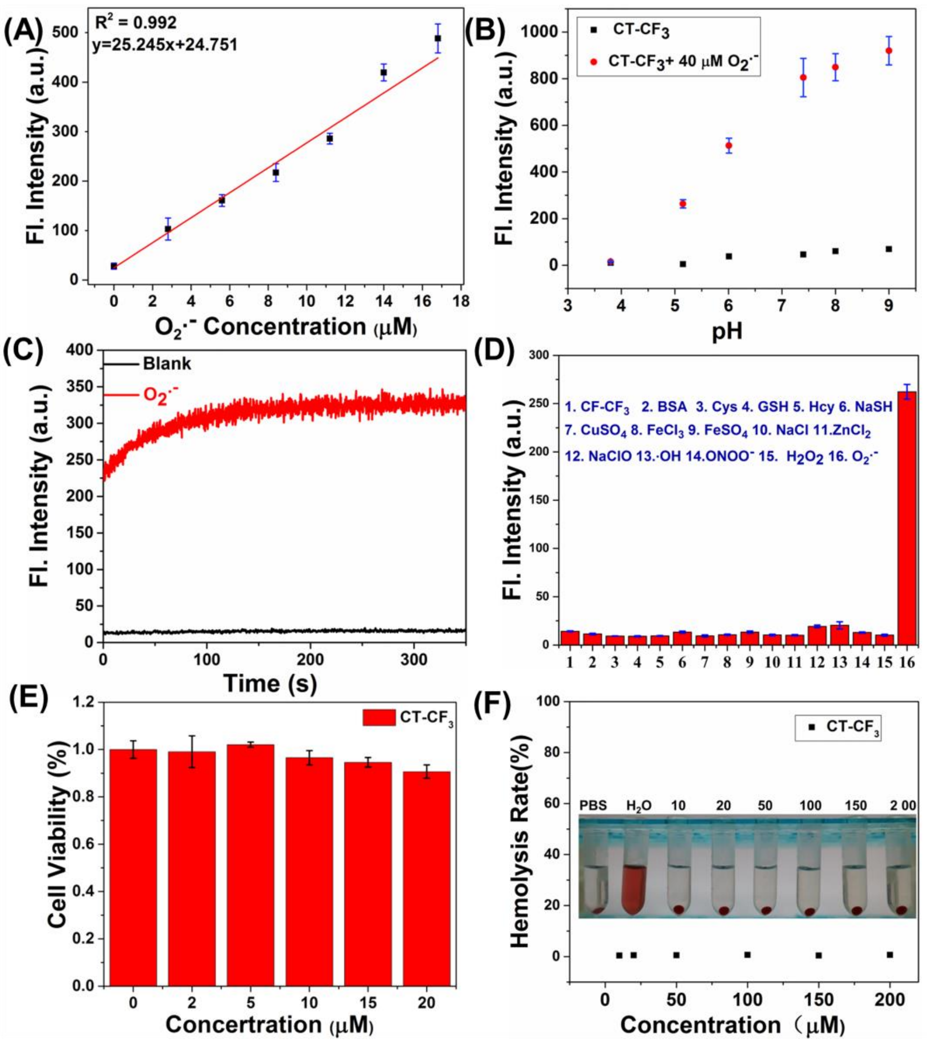

Figure S4 (A) linear relationship between fluorescence intensity at 665 nm and concentrations of $O_2^{\bullet-}$; (B) Fluorescence spectra of CT-CF_3 (5 μM) and CT-CF_3+ $O_2^{\bullet-}$ (40 μM) at different pHs; (C) Time course for changes in the fluorescence of probe CT-CF_3 (5 μM, black curve), and probe CT-CF_3 (5 μM) after the addition of $O_2^{\bullet-}$ (10 μM, red curve). (D) Fluorescence intensity of CT-CF_3 (5 μM) at 665 nm in the presence of various biological species (100 μM), except $O_2^{\bullet-}$ (10 μM), BSA (1 mM), glutathione (1 mM), and cysteine (200 μM). (E) Cytotoxicity of probe CT-CF_3 evaluated on PC-12 cells by MTT assay. (F) Hemolytic analysis of red blood cells treated with different concentration of CT-CF_3. PBS and water as control group for the positive and negative experiments. Insert picture represented the hemolysis result. All spectroscopic data were acquired in phosphate-buffered saline (PBS) buffer (pH 7.4, 30% DMSO) with excitation at 500 nm, the data are presented as mean±SD (n=3).

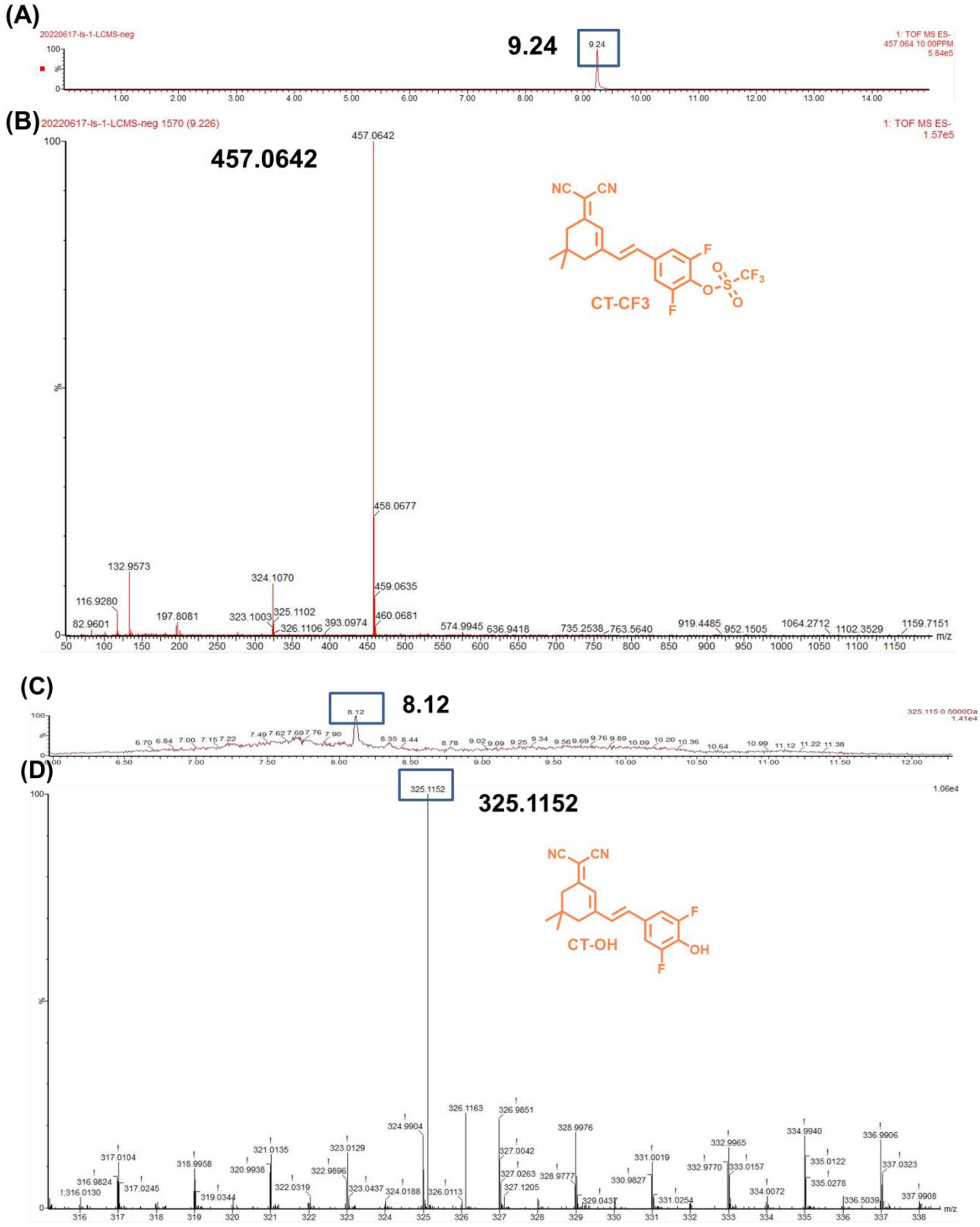

Figure S5 HPLC-MS was used to study the reaction mechanism of CT-CF$_3$ and O$_2^{\bullet-}$. HPLC profiles of CT-CF$_3$ (A) and CT-CF$_3$+KO$_2$ (C). HRMS spectrum of CT-CF$_3$ (B) and CT-CF$_3$+KO$_2$ (D). The retention time of CT-CF$_3$ is 9.24 min and m/z at 457.0642. The retention time of CT-OH is 8.12 min and m/z at 325.1152. The mobile phase for the HPLC was acetonitrile:water (from 5 : 95 to 100 : 0).

Table S2 Pharmacokinetic parameters of CT-CF_3 in plasma after intravenous administration of 2 mg/kg CT-CF_3 in rats (n=3, males)

Group	Analyte	Rat	Gender	AUC_{0-t}	$AUC_{0-\infty}$	$MRT_{0-\infty}$	$t_{1/2}$	CL	Vss
				(h*ng/mL)		(h)	(h)	(mL/min/kg)	(L/kg)
G1-IV-2mg/kg	CT-CF_3	1	M	1417	1426	0.62	0.98	23.4	0.874
		2	M	1356	1364	0.59	0.86	24.5	0.865
		3	M	863	869	0.73	0.80	38.4	1.68
			Mean	1212	1219	0.65	0.88	28.7	1.14
			SD	304	305	0.07	0.09	8.4	0.47

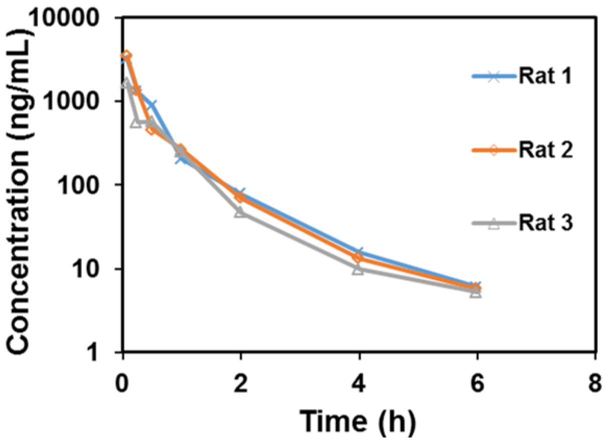

Figure S6 Plasma concentration time curve of CT-CF_3 in plasma after intravenous administration of 2 mg/kg CT-CF_3 in rats.

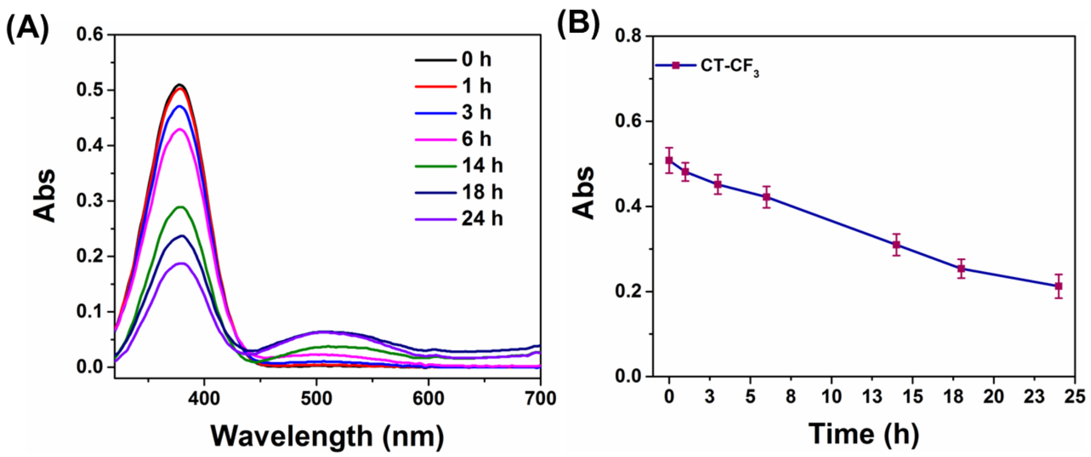

Figure S7 In solution (40% fetal bovine serum + 40% PBS + 20% DMSO) at 37°C, (A) variation of UV-Vis absorption spectrum of 10 μM CT-CF_3 with incubation time; (B) variation of absorbance of CT-CF_3 at 380 nm with incubation time.

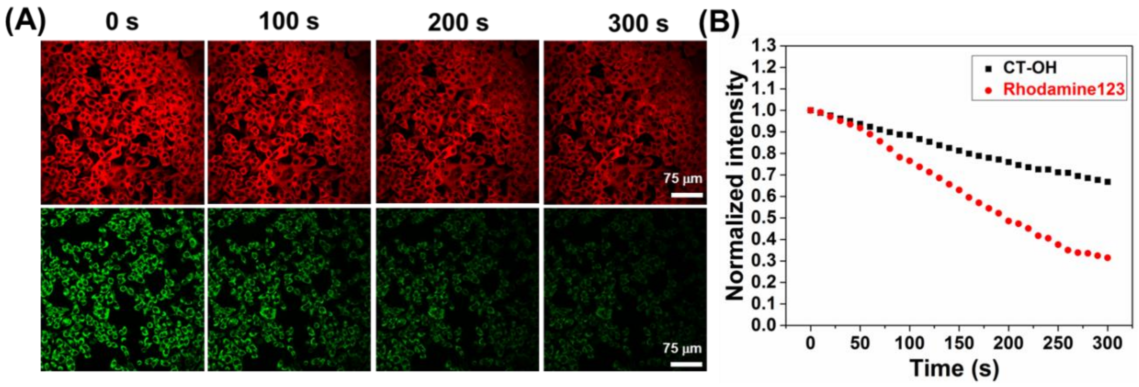

Figure S8 (A) Confocal fluorescence images of CT-OH (10 μM) and Rhodamine 123 (5 μM) in A549 cells under continuous laser irradiation for 300 s, the excitation wavelengths of the red/green channels was 488 nm and the collection wavelength range was 640-720/520-600 nm, scale bar: 75 μm. (B) Variation curve of fluorescence intensity of two probes with laser irradiation time.

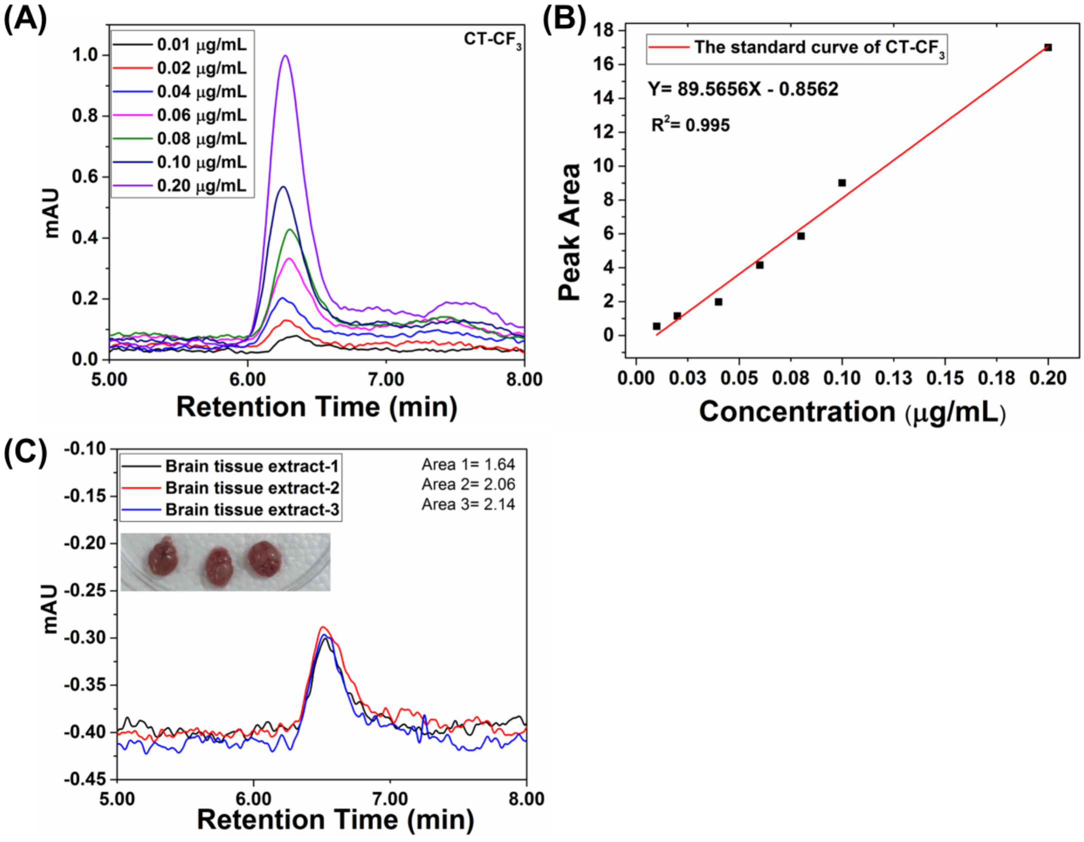

Figure S9 The representative HPLC data of CT-CF_3. (A) HPLC chromatogram curves of different concentrations of CT-CF_3. (B) The standard curves of peak areas of different concentrations of CT-CF_3 in Figure A. (C) HPLC chromatogram of brain homogenates from three mice at 5 min via intravenous injection of CT-CF_3 (0.5mg/kg).

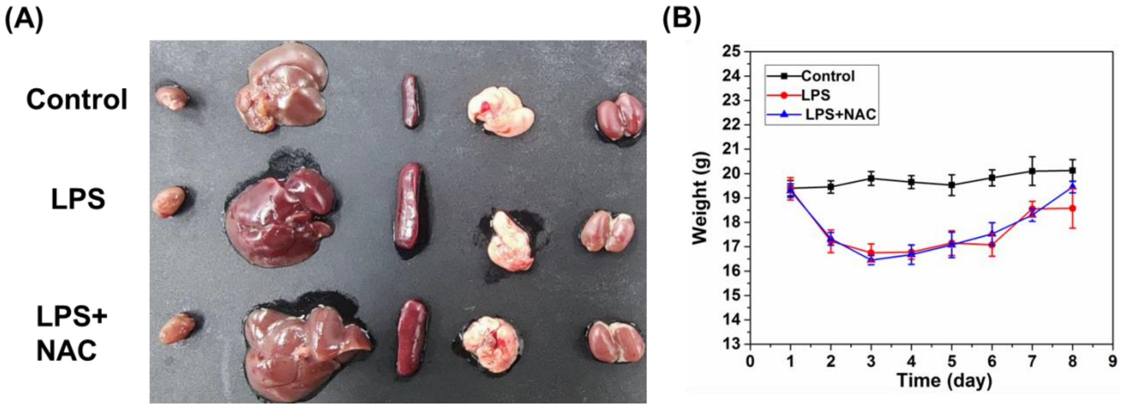

Figure S10 (A) Images of isolated organs from three mice. (B) Body weight (BW) changes of three mice after LPS/NAC administration.

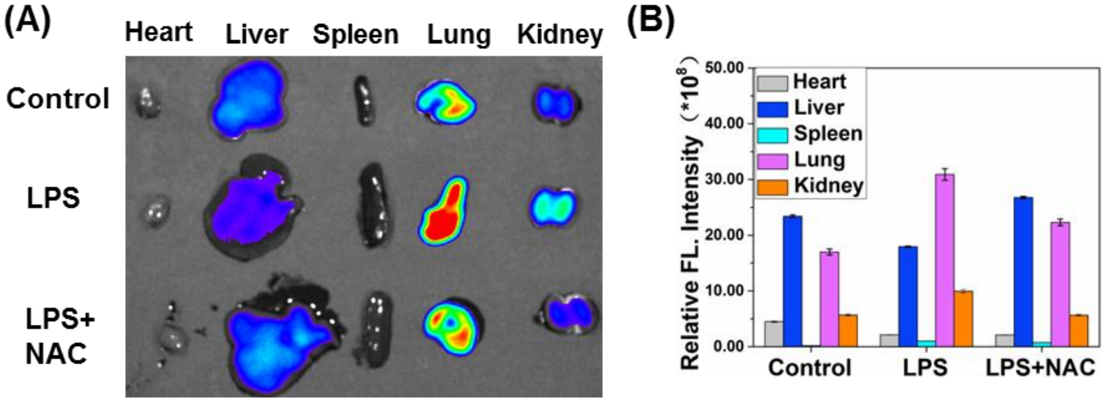

Figure S11 (A) NIR Fluorescence images of isolated organs of the three mice. (B) Quantification of fluorescence intensity in isolated organs of three mice.

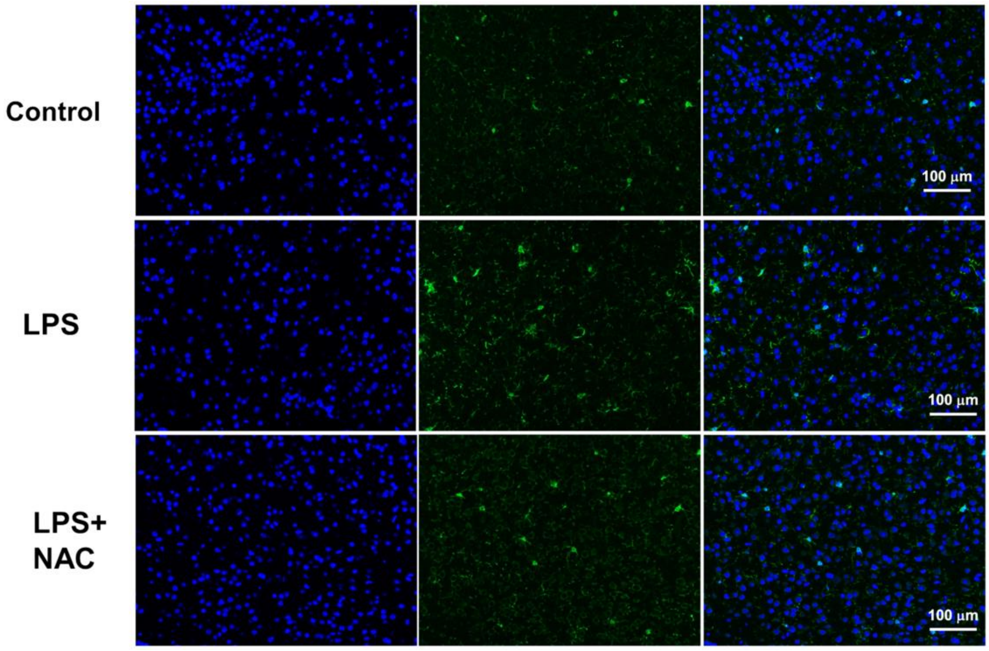

Figure S12 The representative immunofluorescence photographs of microglia in the cortex region of neuroinflammation mice. Iba-1-positive microglia (green) and DAPI-granule cells (blue).

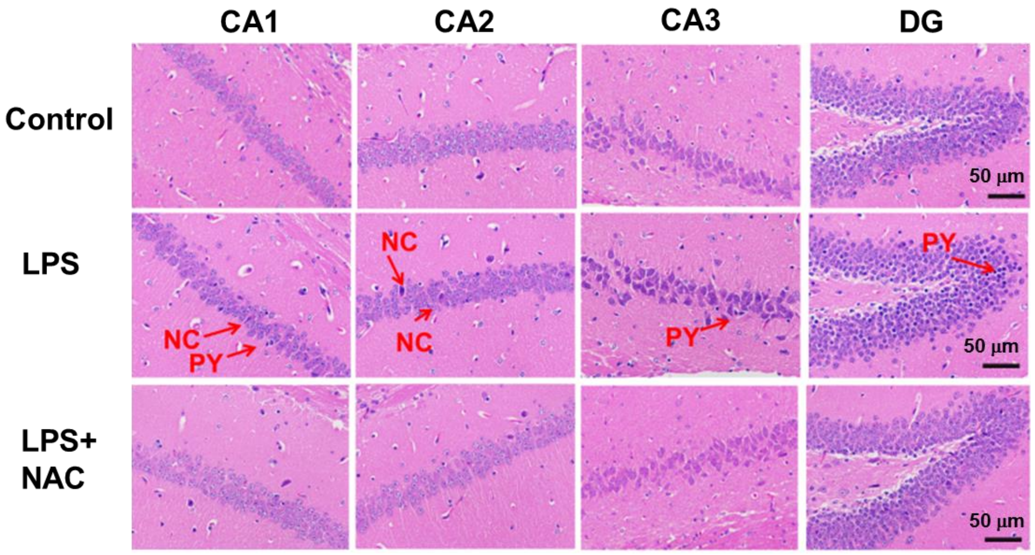

Figure 13 H&E staining for evaluating neuronal damage in the hippocampal region after LPS/NAC administration. Among them, there were pyknosis (PY) and necrosis (NC) of pyramidal neurons and pyknosis (PY) of granulosa cells in the hippocampus of the experimental group (LPS).

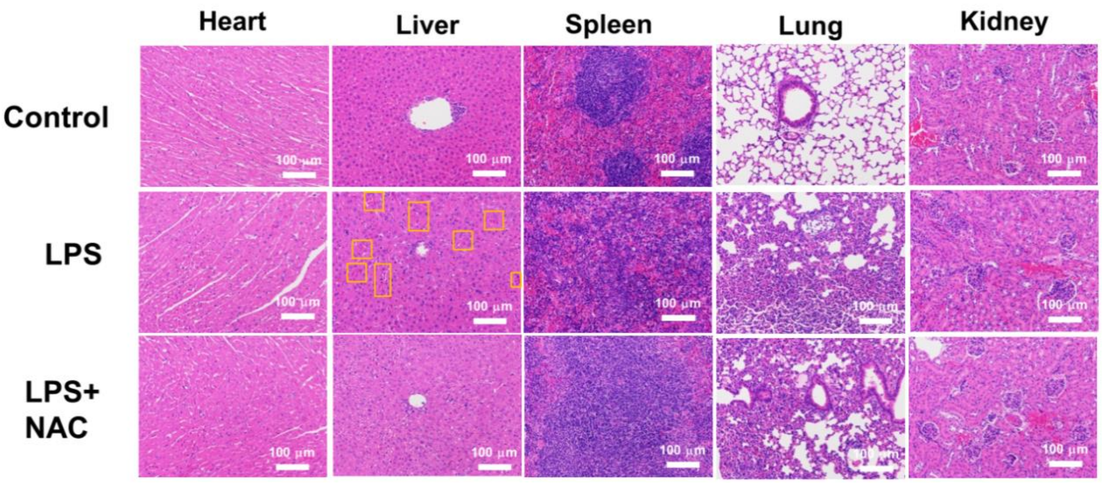

Figure S14 H&E staining of major organs including heart, liver, spleen, lung, and kidney from three mice. The liver, spleen and kidney of the experimental group (LPS) were damaged. Compared to the control group, there were obvious liver damages in the experimental group (LPS): More congestion and hemorrhage spots appear in the liver tissue in the yellow box.

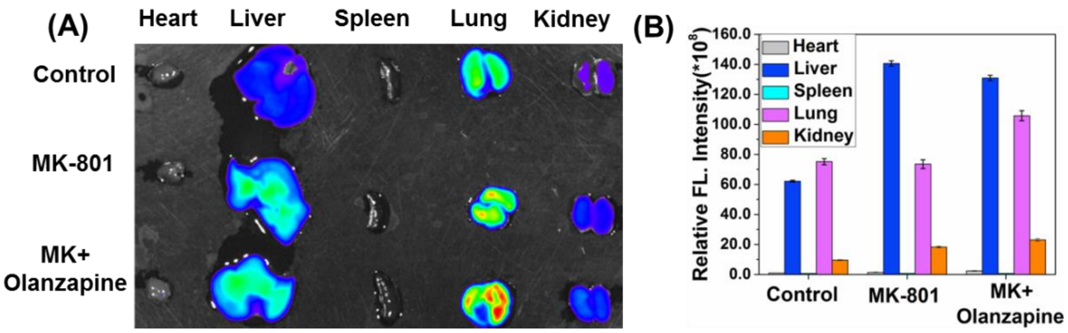

Figure S15 (A) NIR Fluorescence images of isolated organs of the three mice. (B) Quantification of fluorescence intensity in isolated organs of three mice.

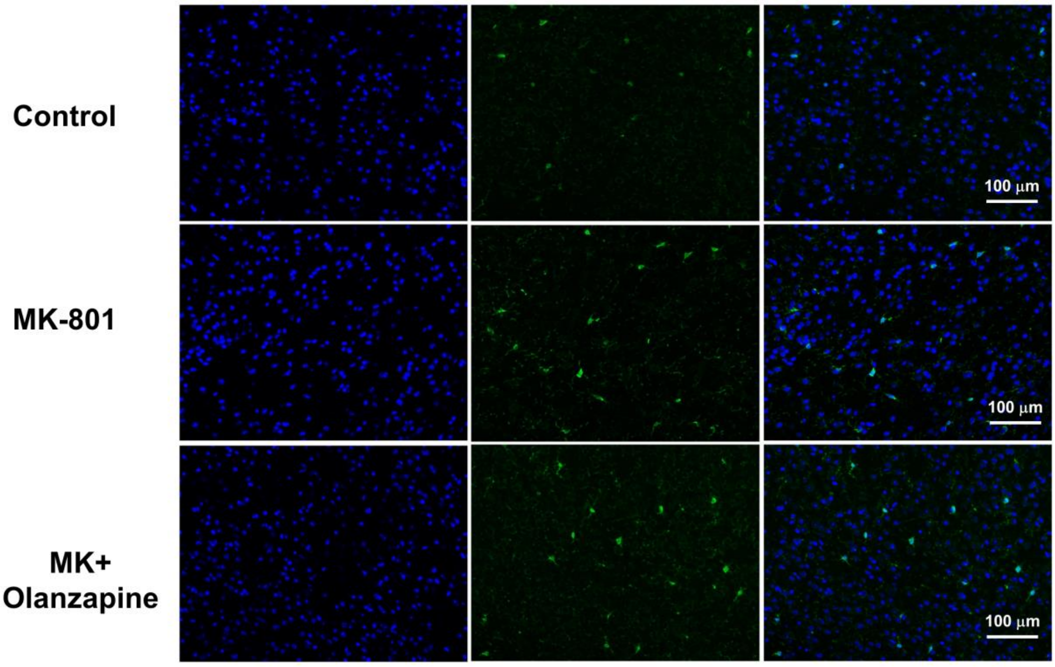

Figure S16 The representative immunofluorescence photographs of microglia in the cortex region. Iba-1-positive microglia (green) and DAPI-granule cells (blue).

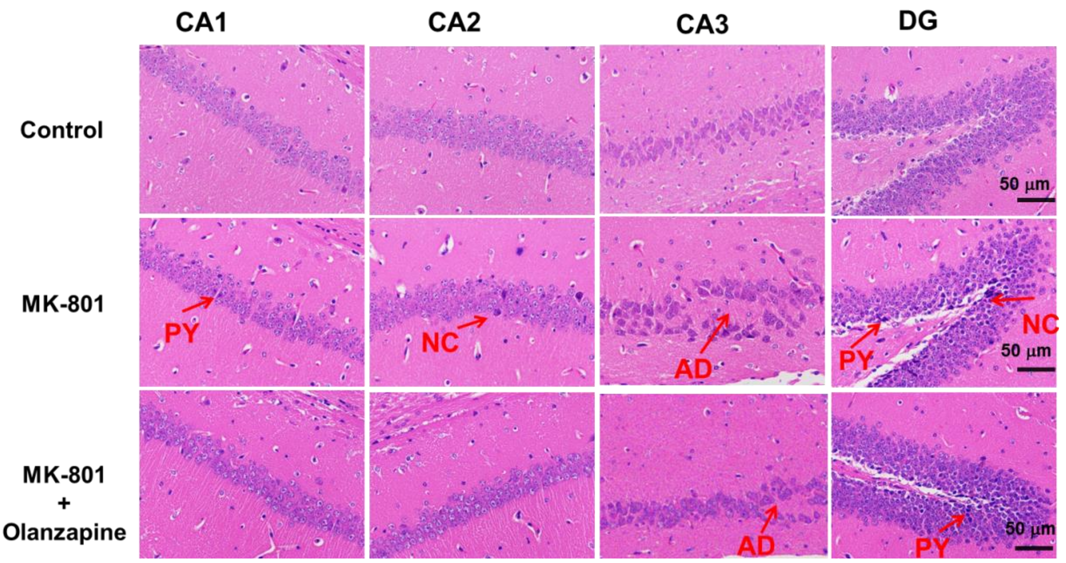

Figure S17 H&E staining for evaluating neuronal damage in the hippocampal region after MK-801/Olanzapine administration. Among them, there were pyknosis (PY), necrosis (NC) and arrangement disorder (AD) of pyramidal neurons and pyknosis (PY) of granulosa cells in the hippocampus of the experimental group (MK-801).

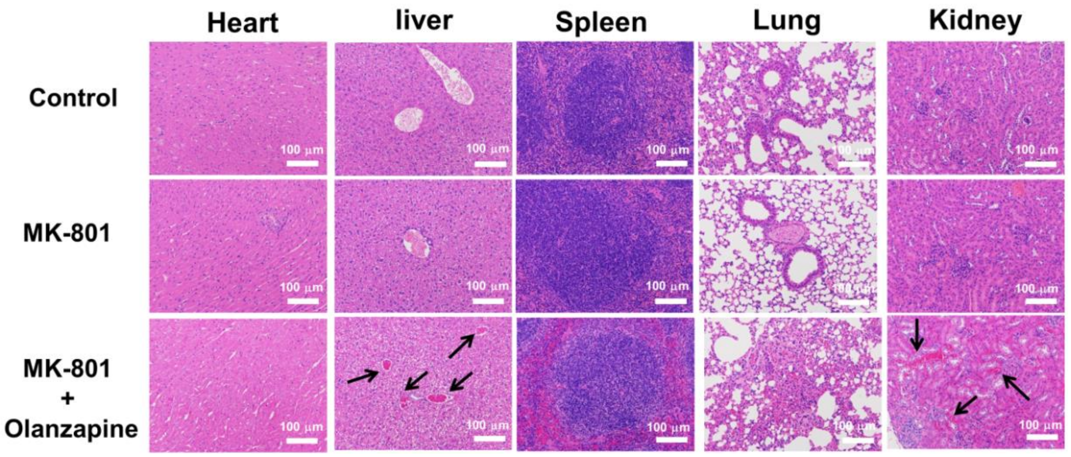

Figure S18 H&E staining of major organs including heart, liver, spleen, lung, and kidney from three mice. Compared to the control group, there were obvious liver and kidney damages in the treatment group: significant congestion and hemorrhage appear in the liver and kidney (black arrow).

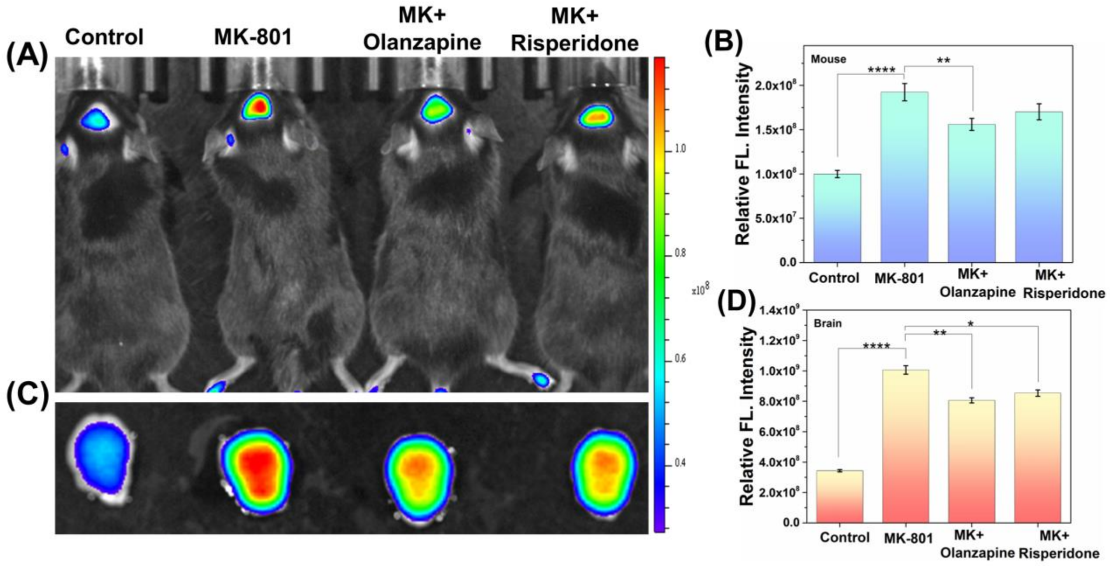

Figure S19 (A) Mapping $O_2^{\bullet-}$ fluxes in the brains of mice with first-onset schizophrenia. Images were recorded after the intravenous (i.v.) injection of $CT\text{-}CF_3$ at 60min. (B) Quantification of the fluorescence intensity of images in (A). (C) NIR Fluorescence images of isolated brains of the four mice. (D) Quantification of the fluorescence intensity of images in (C). Data are represented as the mean±SD (n=3), **** P<0.0001, **** P<0.01 , * P<0. 1.

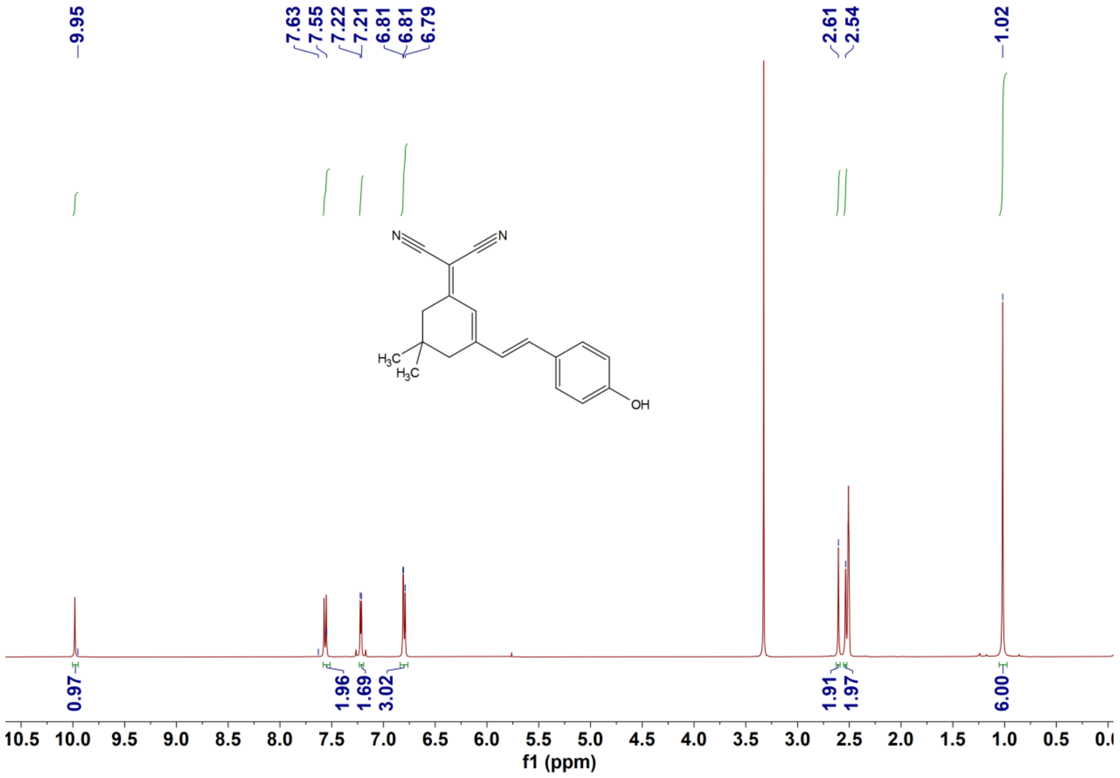

Figure S20 ^{1}H NMR spectrum of CN-OH in DMSO-d6.

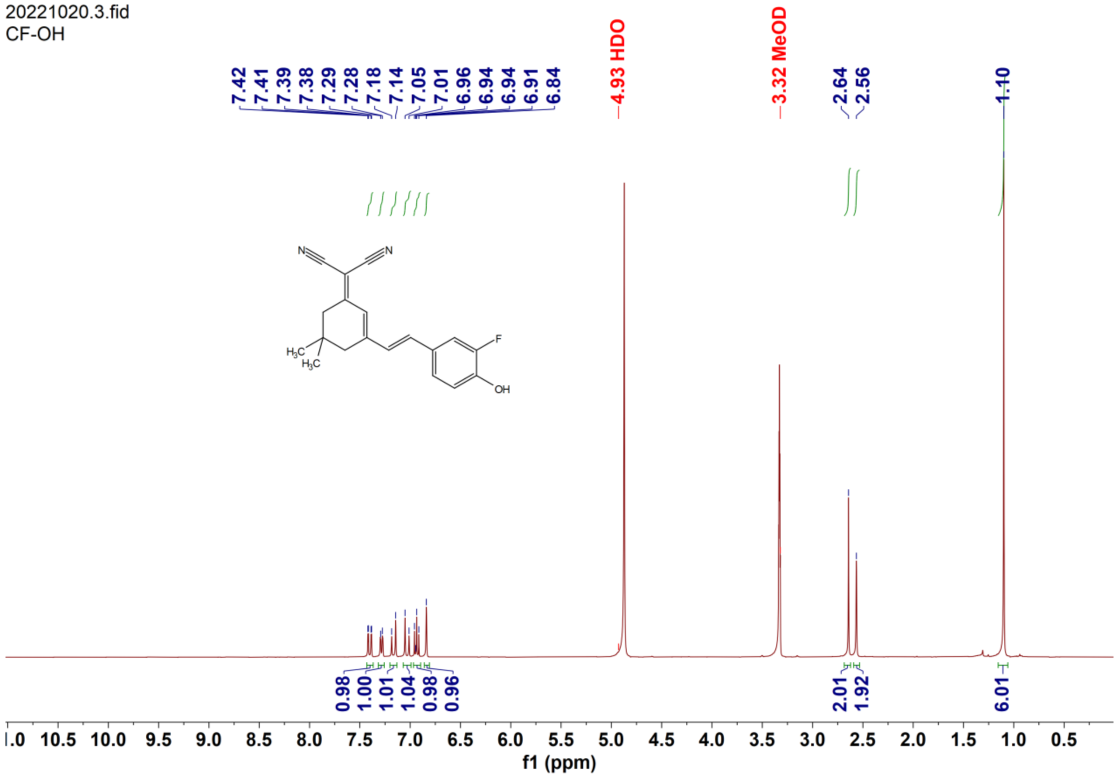

Figure S21 ^{1}H NMR spectrum of CF-OH in MeOD.

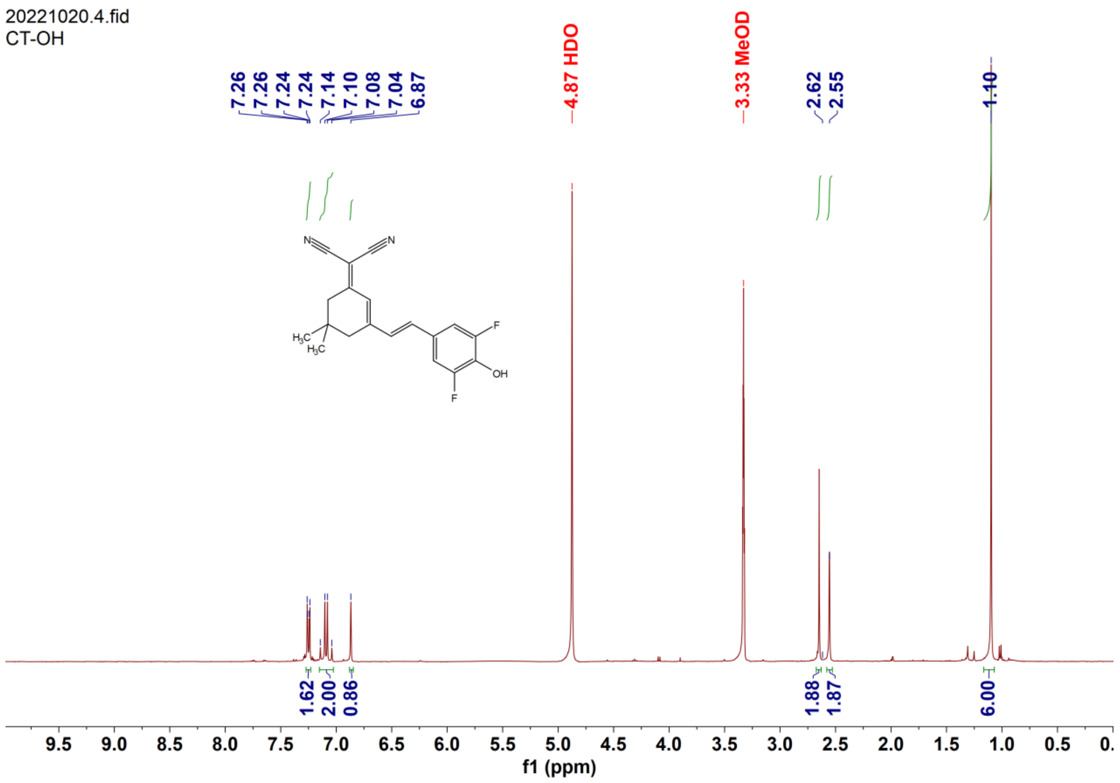

Figure S22 ^{1}H NMR spectrum of CT-OH in MeOD.

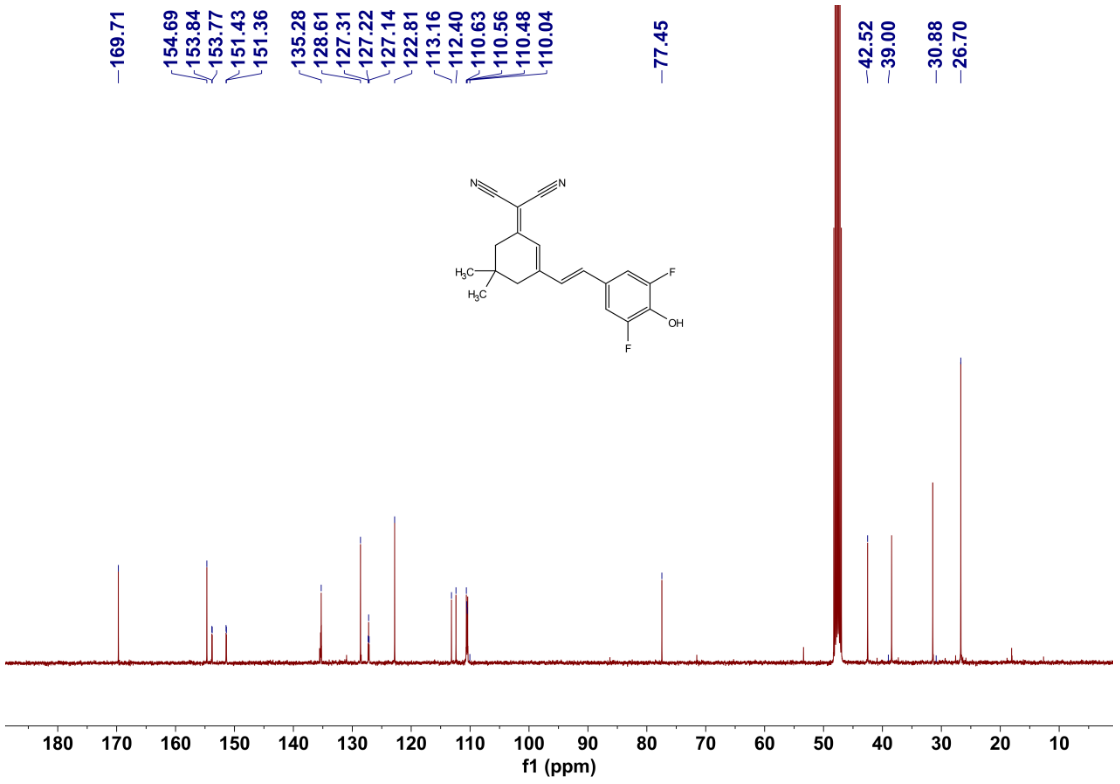

Figure S23 ^{13}C NMR spectrum of CT-OH in MeOD.

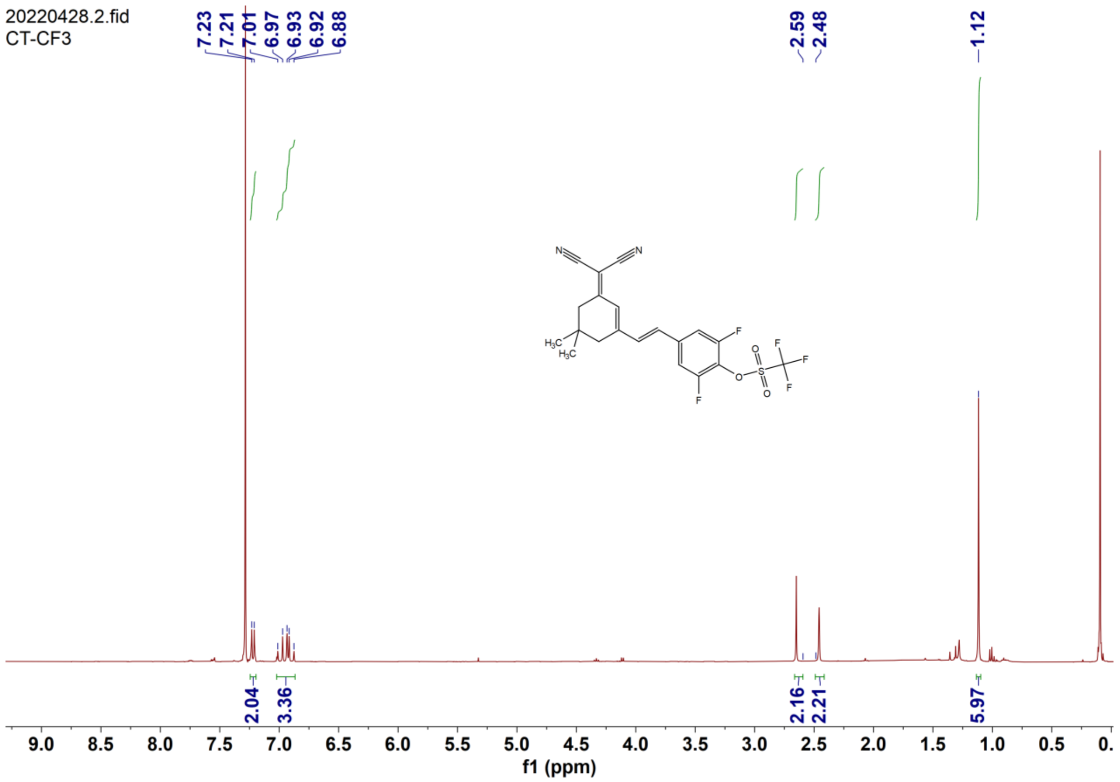

Figure S24 ^{1}H NMR spectrum of CT-CF_3 in $CDCl_3$.

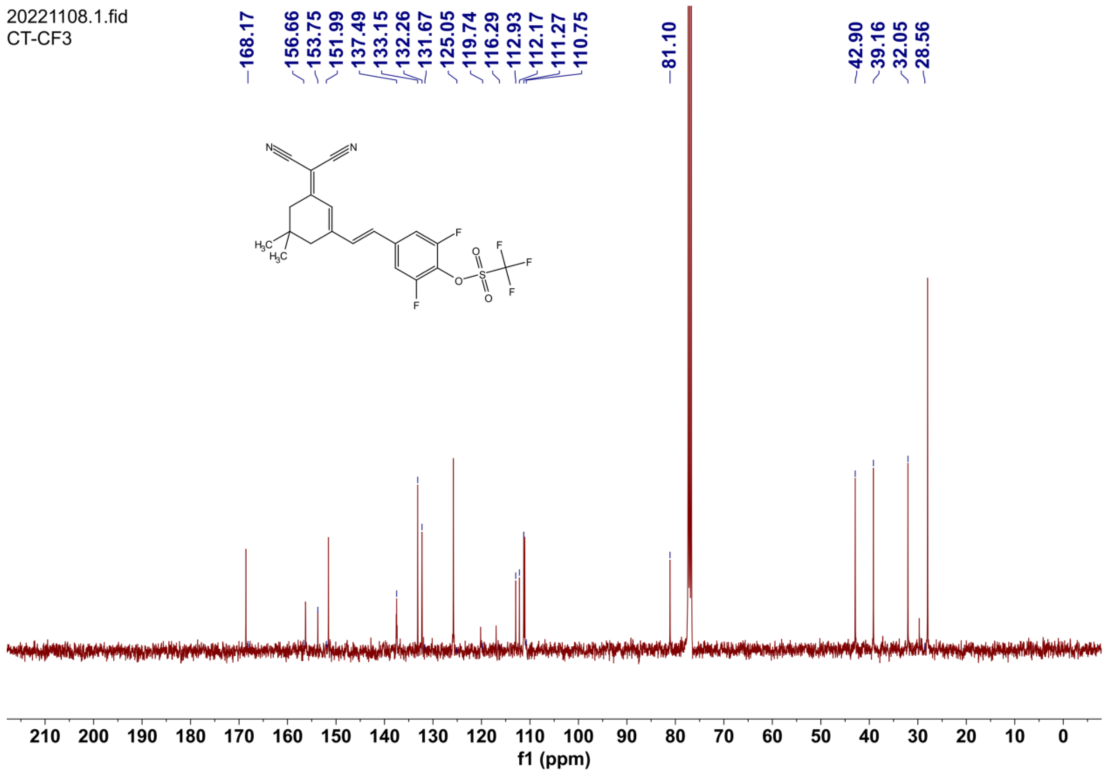

Figure S25 ^{13}C NMR spectrum of CT-CF_3 in $CDCl_3$.

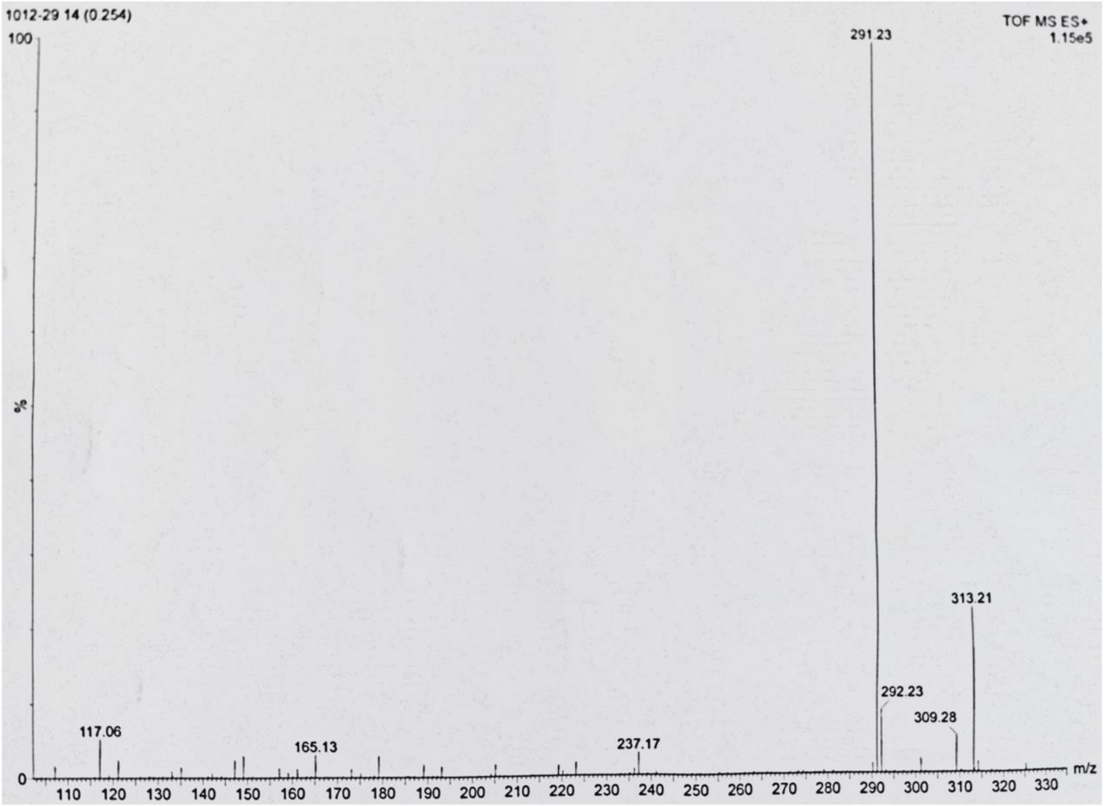

Figure S26 MS spectrum of CN-OH.

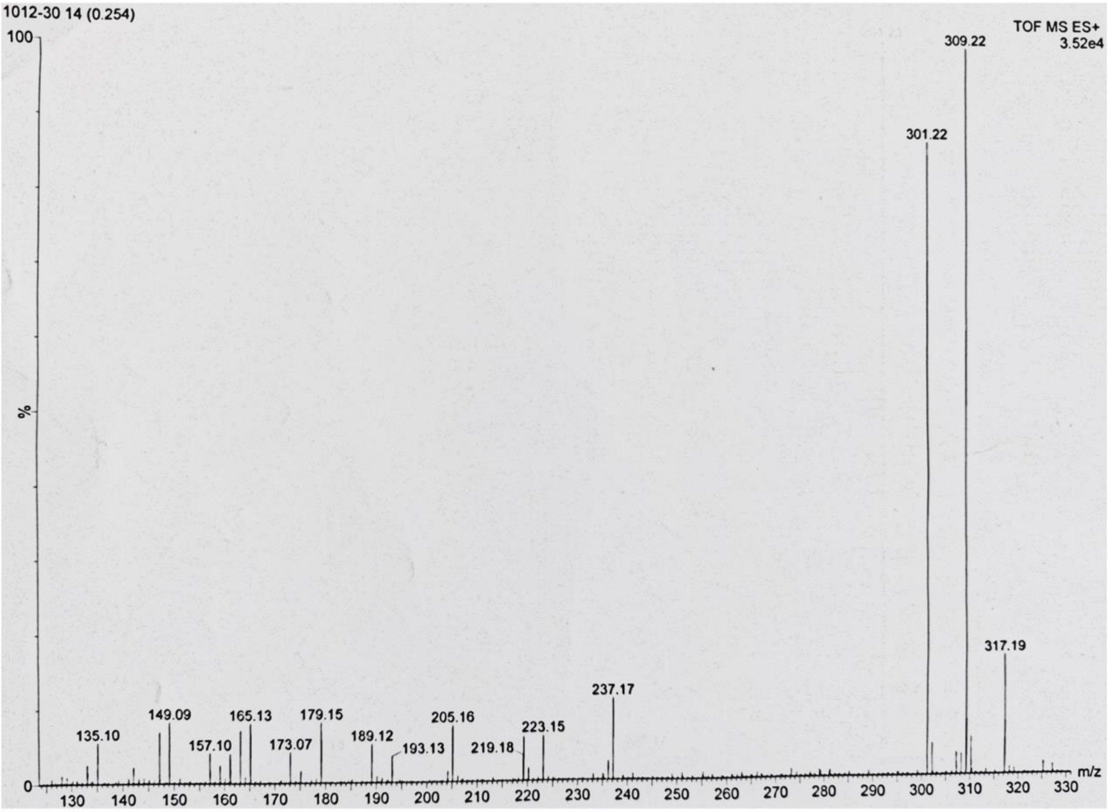

Figure S27 MS spectrum of CF-OH.

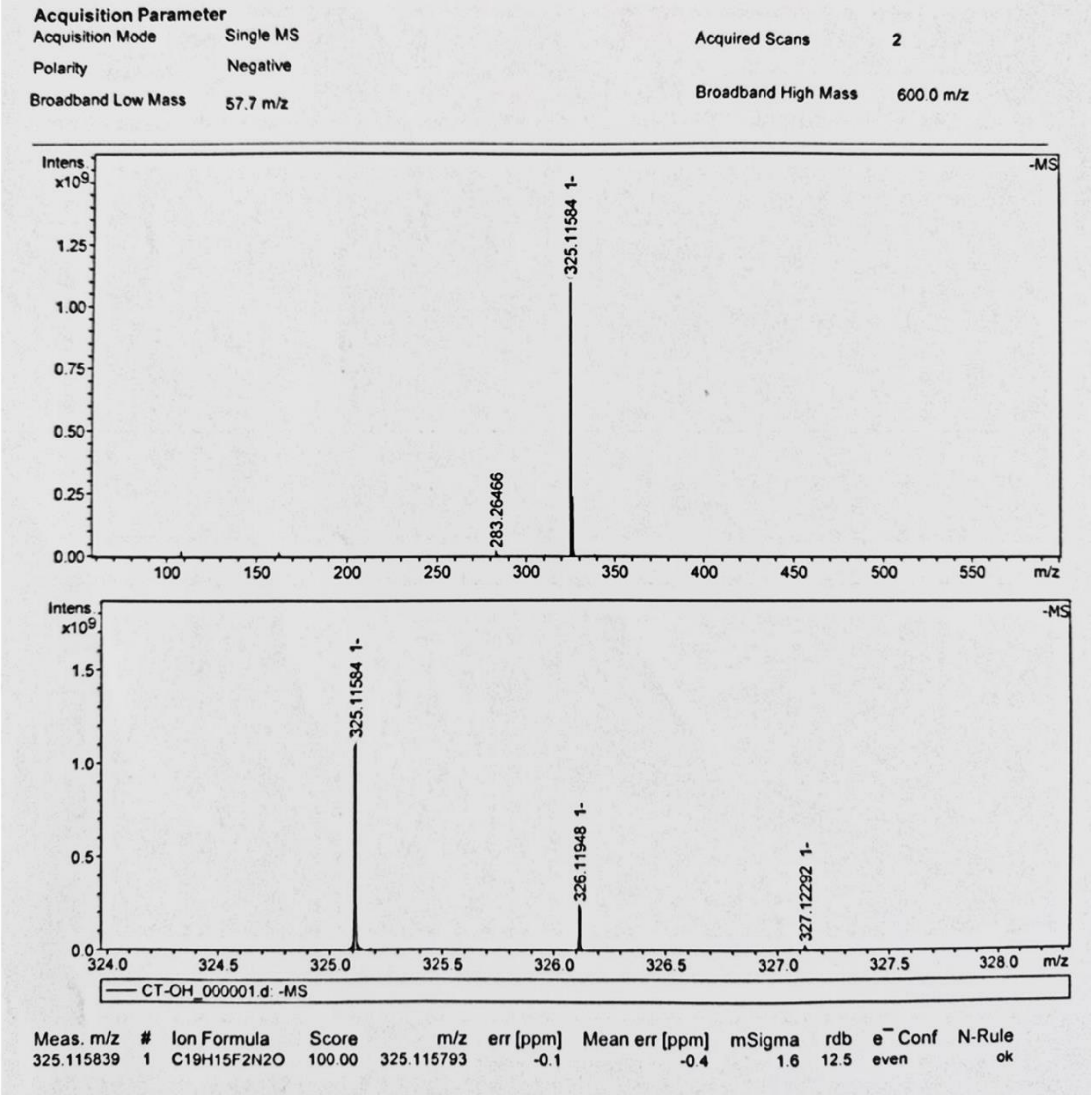

Figure S28 HRMS spectrum of CT-OH.

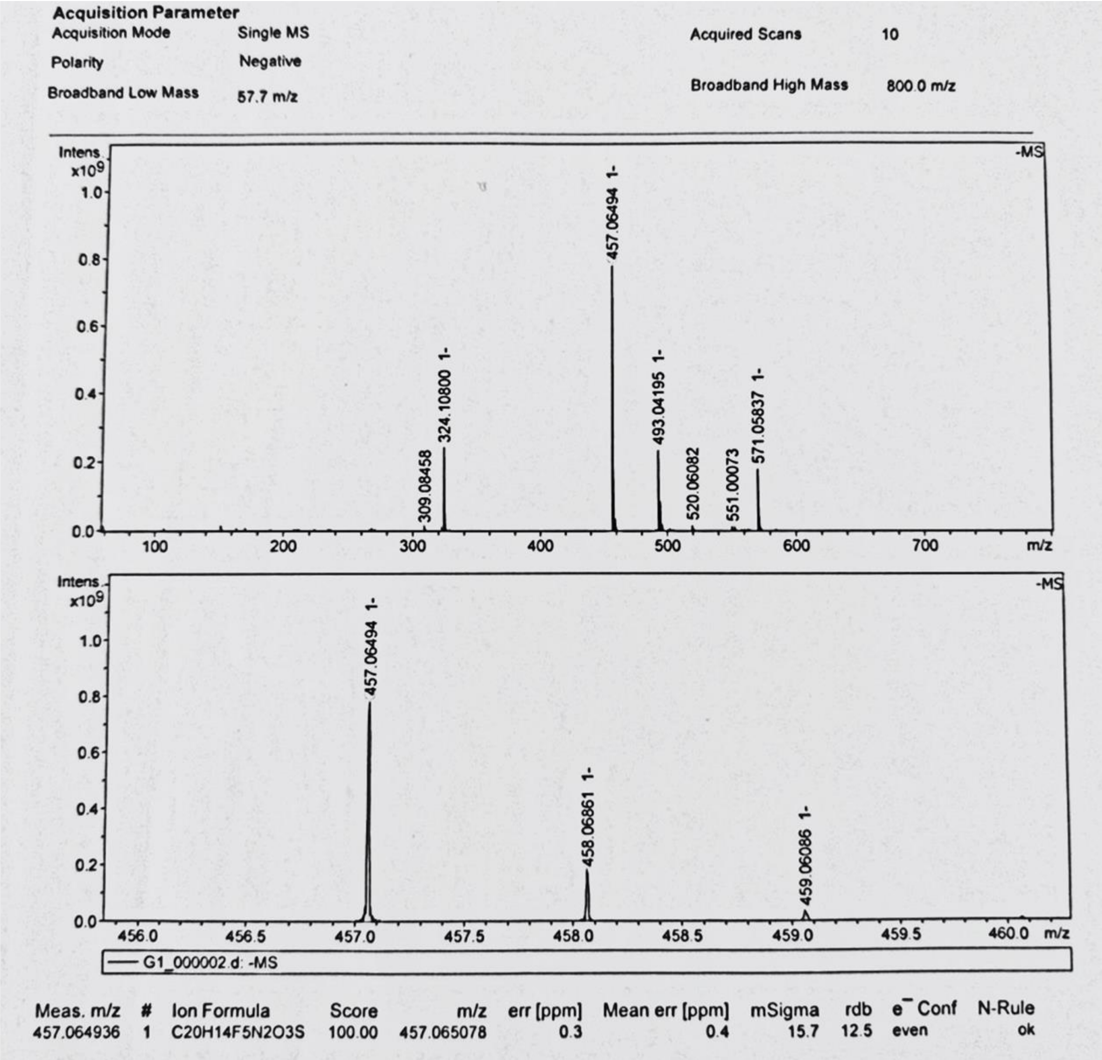

Figure S29 HRMS spectrum of CT-CF_3.

References

[1] L. Wu, J. Liu, X. Tian, R. R. Groleau, S. D. Bull, P. Li, B. Tang and T. D. James, *Chem. Sci*. 2021,**12**, 3921-3928.

[2] M. Ifuku, T. Katafuchi, S. Mawatari, M. Noda, K. Miake, M. Sugiyama and T. Fujino, *J Neuroinflammation* 2012, **9**, 197.

[3] I. Braun, J. Genius, H. Grunze, A. Bender, H. J. Möller, D. Rujescu, *Schizophr. Res*. **2007**, *97*, 254-263.

[4] Y. Xiu, X. Kong, L. Zhang, X. Qiu, Y. Gao, C. Huang, F. Chao, S. Wang, Y. Tang, *J. Psychiatr. Res*. 2015, **63**, 132-140.

[5] J. Yu, D. Qi, M. Xing, R. Li, K. Jiang, Y. Peng, D. Cui, *Brain. Res*. 2011, **1385**, 281-292.
